# Cost‐Effectiveness of Applying Fluoride Varnish to Preschoolers in a Brazilian Scenario: An Economic Modelling Study

**DOI:** 10.1111/cdoe.70031

**Published:** 2025-11-20

**Authors:** Izabel Monteiro Dhyppolito, Rodolfo Castro, Ana Paula Pires dos Santos, Paulo Nadanovsky

**Affiliations:** ^1^ Department of Epidemiology, Institute of Social Medicine Rio de Janeiro State University Rio de Janeiro Brazil; ^2^ Arthur Sá Earp Neto University Center/Medicine Faculty of Petrópolis‐UNIFASE/FMP Petrópolis Brazil; ^3^ Department of Health Administration and Planning National School of Public Health Oswaldo Cruz Foundation Rio de Janeiro Brazil; ^4^ Department of Permanent Education and Integrality in Health Institute of Collective Health, Federal University of the State of Rio de Janeiro Rio de Janeiro Brazil; ^5^ Department of Community and Preventive Dentistry, Faculty of Dentistry Rio de Janeiro State University Rio de Janeiro Brazil; ^6^ Department of Epidemiology and Quantitative Methods in Health, National School of Public Health Oswaldo Cruz Foundation Rio de Janeiro Brazil

**Keywords:** child preschool, cost‐effectiveness analysis, costs and cost analysis, dental caries, fluoride varnish, Markov chains

## Abstract

**Background:**

International economic evaluations have not found convincing evidence that the application of fluoride varnish (FV) in preschool children is a cost‐effective anti‐caries measure, and there is a lack of economic evaluations of FV in the Brazilian context.

**Aim:**

This study evaluated the cost‐effectiveness (CE) of standard care plus FV for Brazilian preschoolers in the general population, comparing it to standard care in terms of prevention of cavitated caries lesions and disability‐adjusted life years (DALY) outcomes.

**Methods:**

Markov models were used, with a 4‐year time horizon and 6‐month cycles. Transition probabilities were obtained from a national epidemiological survey and randomised controlled trials (RCTs). The effectiveness of FV was derived from a systematic review of RCTs. Costs (in Brazilian reais) were sourced from the 2022 National Agency for Supplemental Health dental procedures list. A discount rate of 5% was applied. CE analyses, Markov simulations (MS), and sensitivity analyses (SA) were conducted. Deterministic sensitivity analysis (DSA) used a 95% confidence interval for each variable. For probabilistic sensitivity analysis (PSA), beta distribution curves were used for probabilities, gamma for costs, and lognormal for effectiveness.

**Results:**

Standard care plus FV showed an increase in effectiveness (0.01894 and 0.00018 for avoided caries and DALY, respectively) compared to standard care, with an additional cost of R$131.27 per child in the 4‐year period. The ICER (incremental cost‐effectiveness ratio) was calculated at R$6929.09 per cavitated caries lesion prevented and R$727604.84 per DALY avoided. MS revealed little difference in the percentage of individuals in each health state at the end of the simulations. FV prevented cavities in 4 out of every 100 children over a 4‐year period, at an average annual cost of R$33 per child (assuming each child who developed cavitated caries lesions had only one). DSA indicated that FV effectiveness was the parameter with the highest potential to influence the ICER. PSA suggested high CE thresholds, from which FV would be considered cost‐effective: R$7000 for caries and R$730000 for DALY.

**Conclusion:**

The total cost of care was lower in the group that invested less in prevention (without FV) compared to the group that invested more (with FV). While it is often said that “more prevention is always better,” economic evaluations remind us that not all preventive strategies provide good value for money.

## 1 Introduction

Dental caries represents a significant public health problem due to its high prevalence [1, 2, 3] and its potential negative impact on the quality of life of children and their families. While early stages of dental caries are reversible and do not have a negative impact, more advanced stages, in some children, can lead to pain and functional limitations [4]. In preschool children, caries in its advanced stages may cause pain, difficulty in feeding, interference with learning, and sleep problems [4, 5]. In addition, it results in a substantial economic burden [6, 7].

One of the main recommended strategies for preventing caries in preschool children is the professional application of fluoride varnish (FV) [8, 9] in addition to reducing free sugar intake. Although FV is globally recommended [10], and it is provided in health systems such as the Unified Health System (SUS) in Brazil [9], the benefit conferred by FV is relatively small and questionable [11, 12]. Furthermore, international economic evaluations have not found convincing evidence that the application of FV in preschool children is a cost‐effective measure to prevent caries [13, 14]. Finally, there is a lack of economic evaluations of FV in the Brazilian context.

Given that financial resources are scarce, especially in developing countries, cost‐effectiveness studies can contribute important information for careful resource allocation choices regarding caries prevention. These choices can lead to disinvestment and non‐investment in low‐value oral health care procedures and the reallocation of resources to clearly cost‐effective measures [6, 15].

Therefore, this study aimed to conduct an economic evaluation of professional FV application in preschool children in the Brazilian context. We compared standard care (oral hygiene instruction and dietary counselling provided every 6 months) with standard care plus FV applied during the same visits, within the context of a private clinic charging fees based on the official rates established by the Brazilian National Agency for Supplemental Health (ANS).

## 2 Methods

This is a cost‐effectiveness study based on economic modelling of the Markov model type, reported according to CHEERS guidelines (Consolidated Health Economic Evaluation Reporting Standards) [16] (Appendix S1). The protocol of this study is available at Open Science Framework (https://osf.io/t6gsx/).

### 2.1 Population, Comparators, Context and Setting

The population consists of preschool children (primary dentition), most likely using fluoride toothpaste. The following interventions for caries prevention were considered: standard care (oral hygiene instruction and dietary counselling) every 6 months versus standard care plus FV applied during the same visits. It was assumed that the application of FV is conducted by a dentist in a private clinic setting. The same assumption was made for standard care, provided during routine visits.

### 2.2 Study Perspective, Time Horizon and Cycle Length

The Brazilian National Agency for Supplemental Health (ANS) is the regulatory body overseeing private health insurance and services in Brazil. The perspective of the ANS (specifically the health‐maintaining organisation FioSaúde, sponsored by Fiocruz, a Brazilian public health research institute) was considered, taking into account the direct healthcare costs (professionals, physical infrastructure, supplies) involved in both interventions being compared, as well as the management of carious lesions that would occur within the established time horizon. A time horizon of 4 years was adopted, covering the interval between 2 years of age (the age at which the children enter the model) and 6 years of age; a period that approximately includes the entire primary dentition and precedes the start of mixed dentition. A 6‐month cycle was considered to align with the frequency of FV application recommended in Brazil [9].

### 2.3 Model and Health States

**FIGURE 1:**
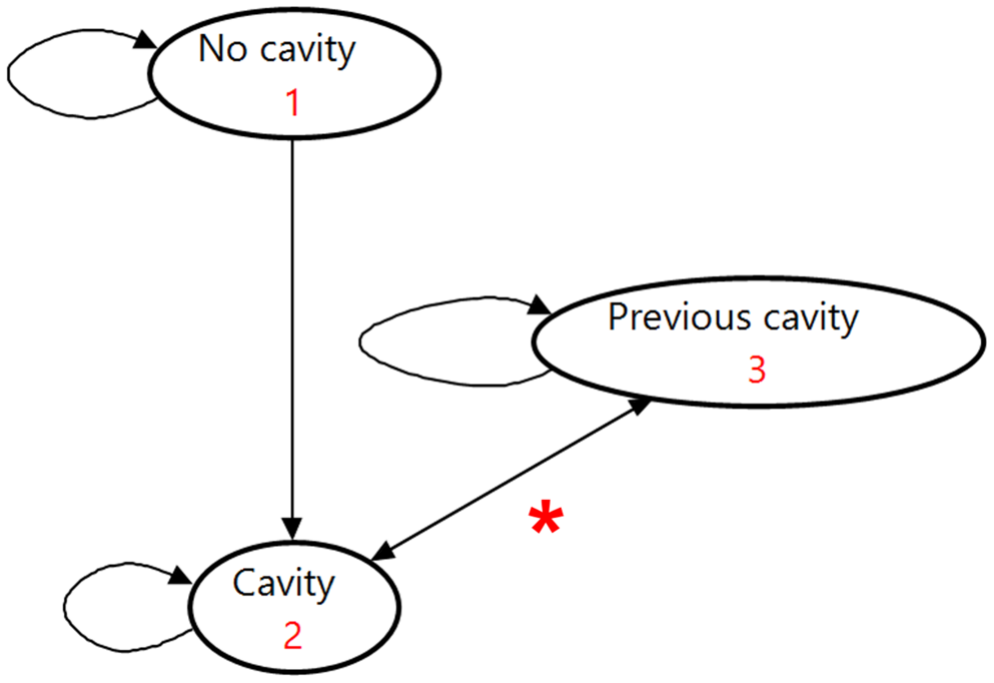
Diagram of the Markov model for the cost‐effectiveness study of the professional application of fluoride varnish to preschoolers in Brazil. Straight arrows represent transitions between states; curved arrows indicate the possibility of remaining in that state at the end of the cycle. Each arrow is associated with different probabilities of transition or permanence within a 6‐month interval. *progression from cavity to previous cavity, that is, a history of open cavity in the past but no cavity at present, can take place in three ways: glass ionomer cement restoration, Hall technique or extraction.

The basic structure of the Markov model is described according to the representation in Figure 1, with the full Markov model tree presented in Appendix S2. The model was developed using TreeAge Pro 2024 R2 (TreeAge Software Incorporated, Williamstown, MA, EUA) [17].

The model in this study considered outcomes at the individual level, assuming that each individual could participate in the modelling by contributing one tooth at a time. The outcome considered was the cost per cavity avoided (we assumed that each child who developed cavitated caries lesions had only one). In addition, data on DALYs (disability‐adjusted life years) related to caries in the primary teeth were used to obtain the cost per DALY avoided. The DALY weight applied (0.01) reflects the average health loss associated with a cavitated caries lesion in a primary tooth, as derived from the Global Burden of Disease Study 2019. This value represents an estimation of the health burden experienced by a child during the period in which the carious lesion is present, acknowledging that the exact duration and severity may vary [18]. This approach allows for a standardised metric to compare the health impact of caries within economic evaluations.

### 2.4 Transition Probability and Effectiveness

The transition probabilities of the model were calculated from national epidemiological survey databases (SB Brasil) [1] and clinical trials [19, 20] (Table 1). The data from this epidemiological survey were processed using the R program (with RStudio interface 2022.02.3 Build 492. PBC, all rights reserved) and the epiDisplay package. The designation of the type of treatment to be received was based on the codes from the SB Brasil database. For decayed teeth coded as requiring restoration of one, two, or more surfaces, restorative treatment with glass ionomer cement was assumed. For decayed teeth coded as requiring a crown, treatment using the Hall Technique was assumed.

**TABLE 1:** Probability, effectiveness and cost parameters used in the cost‐effectiveness analysis of fluoride varnish for preschoolers in a Brazilian dental setting.

	Parameter	Baseline scenario	Change		Distribution	Source
			Lower limit	Upper limit		
Standard care (probabilities)	Probability of progressing from the state “*no cavity”* to *“cavity”*	0.06873	0.0665	0.071	Beta	SB Brasil 2010 [1]
	Probability of remaining in the state *“no cavity”*	#	#	#	Beta	SB Brasil 2010 [1]
	Probability of progressing from the state *“cavity”* to glass ionomer cement	0.873	0.869	0.877	Beta	SB Brasil 2010 [1]
	Probability of progressing from the state *“cavity”* to hall technique restoration	0.004	0.003	0.005	Beta	SB Brasil 2010 [1]
	Probability of progressing from the state *“cavity”* to Tooth Extraction	#	#	#	Beta	SB Brasil 2010 [1]
	Probability of a successful glass ionomer cement and evolution to a “previous cavity” state	0.63	0.49	0.74	Beta	Supplementary File 7 of Araújo et al., 2020 [19]
	Probability of glass ionomer cement failure and evolution to “cavity” state	#	#	#	Beta	Araújo et al., 2020 (additional file 7) [19]
	Probability of hall technique success and evolution to “previous cavity” state	0.98	0.88	0.99	Beta	Araújo et al., 2020 (additional file 7) [19]
	Probability of hall technique failure and consequent evolution to “cavity” state	#	#	#	Beta	Araújo et al., 2020 (additional file 7) [19]
	Probability of remaining in the “previous cavity” state	#	#	#	Beta	Guedes et al., 2017 [18]
	Probability of progressing from “previous cavity” to “cavity” state	0.28	0.14	0.58	Beta	Guedes et al., 2017 [20]
FV (probabilities)	Probability of progressing from the state “*no cavity”* to *“cavity”*	p_nocav_cavity * eff_rr = 0.06873 * 0.88 = 0.0604824	0.0586	0.0625	Beta	SB Brasil 2010 [1]; de Sousa FSO et al., 2019 [11]
	Probability of remaining in the state *“no cavity”*	#	#	#	Beta	SB Brasil 2010 [1]
	Probability of progressing from the state *“cavity”* to glass ionomer cement	0.873	0.869	0.877	Beta	SB Brasil 2010 [1]
	Probability of progressing from the state *“cavity”* to hall technique restoration	0.004	0.003	0.005	Beta	SB Brasil 2010 [1]
	Probability of progressing from the state *“cavity”* to Tooth Extraction	#	#	#	Beta	SB Brasil 2010 [1]
	Probability of a successful glass ionomer cement and evolution to a “previous cavity” state	#	#	#	Beta	Araújo et al., 2020 (additional file 7) [19]
	Probability of glass glass ionomer cement failure and evolution to “cavity” state	eff_rr*0.37 = 0.3256	0.2288	0.4488	Beta	Araújo et al., 2020 (additional file 7) [19, 21]; de Sousa FSO et al., 2019 [11]
	Probability of hall technique success and evolution to “previous cavity” state	#	#	#	Beta	Araújo et al., 2020 (additional file 7) [19]
	Probability of hall technique failure and consequent evolution to “cavity” state	eff_rr*0.02 = 0.0176	0.0088	0.1056	Beta	Araújo et al., 2020 (additional file 7) [19]; de Sousa FSO et al., 2019 [11]
	Probability of remaining in the “previous cavity” state	#	#	#	Beta	Guedes et al., 2017 [20]
	Probability of progressing from “previous cavity” to “cavity” state	eff_rr*0.28 = 0.2464	0.1232	0.5104	Beta	Guedes et al., 2017 [20]; de Sousa FSO et al., 2019 [11]
	FV effectiveness *	0.88	0.81	0.95	LogNormal	de Sousa FSO et al., 2019 [11]
Costs **	Costs related to “no cavity” and “previous cavity” states	118.50	142.2	94.8	Gamma	Operadora FioSaúde (Appendix S3)
	Costs related to “cavity” state	118.50	142.2	94.8	Gamma	Operadora FioSaúde (Appendix S3)
	Cost related to glass ionomer restorations	179.50	215.4	143.6	Gamma	Operadora FioSaúde (Appendix S3)
	Cost related to Hall Technique restorations	254.50	305.4	203.6	Gamma	Operadora FioSaúde (Appendix S3 and S4)
	Cost related to tooth extraction	172.50	207	138	Gamma	Operadora FioSaúde (Appendix S3)
	Cost related to FV application on both arches	78.00	93.6	62.4	Gamma	Operadora FioSaúde (Appendix S3)

*Note:* All values are adjusted for a cycle length of 6 months. (#) indicates complementary probabilities calculated by the program, based on the premise that the sum of the probabilities of complementary outcomes must be equal to one. * FV effectiveness refers to the relative risk of a child developing at least one cavity when FV is professionally applied in addition to standard care, compared to standard care. ** Costs in Brazilian Reais. On 20 of August 2024 1 US Dollar could buy 5.5 Reais.

The effectiveness of FV was obtained from a systematic review with meta‐analysis on the effectiveness of FV in preschoolers [11], which estimated a pooled relative risk of 0.88 (95% confidence interval 0.81–0.95). These data were used as an adjustment factor, applied as a multiplier to the transition probabilities already adapted to the 6‐month cycle duration in the model.

### 2.5 Costs, Currency and Discount Rate

Cost data related to the procedures list of the National Agency for Supplemental Health (ANS) with descriptions by codes from the Unified Supplemental Health Terminology (TUSS) obtained from the FioSaúde were used (Appendix S3). The procedures considered for cost estimates from the FioSaúde database and their associated costs are detailed in Appendices III and IV.

Costs were considered in Reais, Brazilian currency, in the year 2022. An annual discount rate of 5% was applied to both health costs and outcomes, as recommended in methodological guidelines for economic evaluations [22].

### 2.6 Willingness to Pay

For exploratory analysis, we adopted willingness‐to‐pay thresholds based on an official Brazilian recommendation for the DALY model and on a previous study conducted in Brazil for the caries cavity model. A willingness to pay of approximately R$40,000 (equivalent to 1 GDP per capita) per QALY (Quality‐adjusted life year), a recommendation recently approved by CONITEC (National Committee for Health Technology Incorporation in the Brazilian Public Health System) [21], was considered for the DALY outcome. For the outcome of cost per cavity avoided, an average willingness to pay of R$60 (approximately US$10, based on the 2024 exchange rate), obtained from a Brazilian study, was adopted, reflecting the perspective of private payers [23].

### 2.7 Cost‐Effectiveness Analysis

The total cost and effectiveness of each intervention were calculated through modelling in TreeAge Pro Healthcare 2024 R2.1 (TreeAge Software LLC, Williamstown, MA, USA). Markov cohort simulations were also performed considering standard care and FV to compare the percentage difference of individuals remaining in the non‐cavitated, cavitated, and previous caries health states over each cycle and at the end of the model.

### 2.8 Sensitivity Analysis

Two types of sensitivity analysis were carried out: deterministic and probabilistic (considering both cavitated caries and DALY avoided as outcomes) using TreeAge Pro Healthcare 2024 R2 software (TreeAge Software LLC, Williamstown, MA, USA). The deterministic analysis evaluates a single parameter by altering its values within a predetermined confidence interval. In contrast, the probabilistic analysis considers all model parameters simultaneously, with value variations following the distribution curves adjusted for each variable.

In the deterministic sensitivity analysis, upper and lower limits of the 95% confidence interval for the probabilities of success and failure of glass ionomer and Hall Technique were obtained from a randomised controlled trial [19]. For the other probabilities in the model, 95% confidence intervals were calculated in the R program (RStudio 2022.02.3 Build 492. PBC, all rights reserved) with the aid of the epiDisplay package. For costs, an uncertainty of 20% was considered for the upper and lower limits around the point measurement. Finally, with regard to the effectiveness of FV, we considered the 95% confidence interval of the relative risk (0.81 to 0.95) estimated in a systematic review with meta‐analysis [24].

Probabilistic sensitivity analyses were conducted using Monte Carlo simulations with 1000 iterations. For the probability parameters, beta distribution curves [25] were considered, adjusting the curves based on the standard deviations of the model parameters. For FV effectiveness, we used the LogNormal distribution curve [25] adjusted using data extracted from the source systematic review [11]. Finally, for the cost parameters, the gamma distribution curve was considered, which was adjusted assuming standard deviations equal to 20% of the point measure.

### 2.9 Assumptions

The following assumptions were made: caries risk does not vary over time; FV does not cause significant adverse effects [11]; the simulation began with 2‐year‐old children and ended at 6 years of age (starting about when most of the children usually have the full primary dentition and ending about when permanent teeth erupt); the SB Brasil data at 5 years refer to the age of 5 years and 11 months, totaling 71 months. From this total, 12 months were subtracted, corresponding to the first year of life of the children, a period assumed to be without teeth or with incisors only, and therefore not at risk of developing caries; all children enter the model in a caries‐free state (no cavities); every cavitated caries lesion will receive some type of treatment, whether restorative (glass ionomer or hall technique) or extraction, and that this restoration will be susceptible to failure, which will result in the individual's transition to a cavitated state; the unit of measurement is the child, as the effectiveness estimate was derived from studies assessing the risk of a child developing at least one cavity. For the current cost‐effectiveness model, we assumed that each child who developed cavities had only one; children most likely use fluoride toothpaste.

To increase transparency and credibility, the model underwent an iterative development and validation process. A multidisciplinary team, including three experienced paediatric dentistry clinicians and researchers with expertise in epidemiology and evidence‐based dentistry, participated in several meetings to review the model's structure, assumptions, and parameters, establishing face validity by confirming the model's capacity to realistically simulate the prevention of dental caries with or without fluoride varnish. The model was further compared with published economic models on fluoride varnish interventions, ensuring consistency with existing literature and clinical expectations. Although external validation with independent datasets was limited due to data constraints, this systematic refinement and expert review process aimed to maximise model validity and reliability.

## 3 Results

The cost‐effectiveness analysis of standard care compared to standard care plus FV application in preschoolers, considering both outcomes, caries and DALY, is shown in Table 2.

**TABLE 2:** Cost‐effectiveness analysis comparing standard care plus fluoride varnish application with standard care.

Outcome	Dominance^a^	Strategy	Accumulated cost in the 4‐year period^b^	Incremental cost^c^	Effectiveness^d^	Incremental effectiveness^e^	ICER^f^
Cavity avoided	No dominance	Standard care	203.40853		1.5474046		
	No dominance	Standard care plus FV	334.67498	131.26645	1.5663489	0.01894424	6929.095
DALY avoided	No dominance	Standard care	203.408527		0.0013445		
	No dominance	Standard care plus FV	334.674981	131.26645	0.0011641	0.00018041	727604.842

^a^ Refers to whether one strategy is clearly superior to another. “No dominance” indicates that neither strategy is clearly superior.

^b^ Costs in Brazilian Reais. On 20 August 2024 1 US Dollar could buy 5.5 Reais.

^c^ Difference in cost between two strategies.

^d^ Represents cavities avoided or DALYs (Disability‐Adjusted Life Years) avoided per child over the intervention period.

^e^ Difference in health benefit between two strategies.

^f^ Calculated by dividing the incremental cost by the incremental effectiveness.

The data shown in Table 2 indicate that “standard care plus FV” has higher costs (an increase of R$131.27) and greater effectiveness than “standard care” (an increase of 0.0189). The ICER of 6929.0954 represents the cost per cavity avoided within the proposed time horizon. As for the DALY outcome, “standard care plus FV” has higher costs (an increase of R$131.27) and lower effectiveness (which is desirable considering that DALY is a negative outcome) than “standard care” (a small increase of 0.00018). The ICER of 727604.84 represents the cost per DALY avoided within the proposed time horizon.

The results of the Markov analysis over a 4‐year time horizon, which simulates a cohort for standard care and a cohort for standard care plus FV, indicate that about 57% of individuals would remain in the ‘non‐cavitated’ state in the standard care group, while about 61% would remain in this state in the standard care plus FV group. Regarding the ‘cavitated’ state, approximately 16% of individuals in the standard care group would be in this state compared to 13% in the standard care plus FV group.

Evaluating the ‘previous cavity’ state, which represents a history of the disease in the past but no cavity at present, about 28% of individuals would be in this state in the standard care group and 26% in the standard care plus FV group at the end of eight cycles. In other words, the application of FV allowed more children to remain caries‐free and minimized the percentage of children ending up in the active disease state or in a healthy state but with past caries experience.

The average cost per child over the 4‐year period was R$334 in the standard care plus FV group and R$203 in the standard care group (Table 2), resulting in a difference of R$131. This translates to an additional average annual cost of R$33 per child.

**FIGURE 2:**
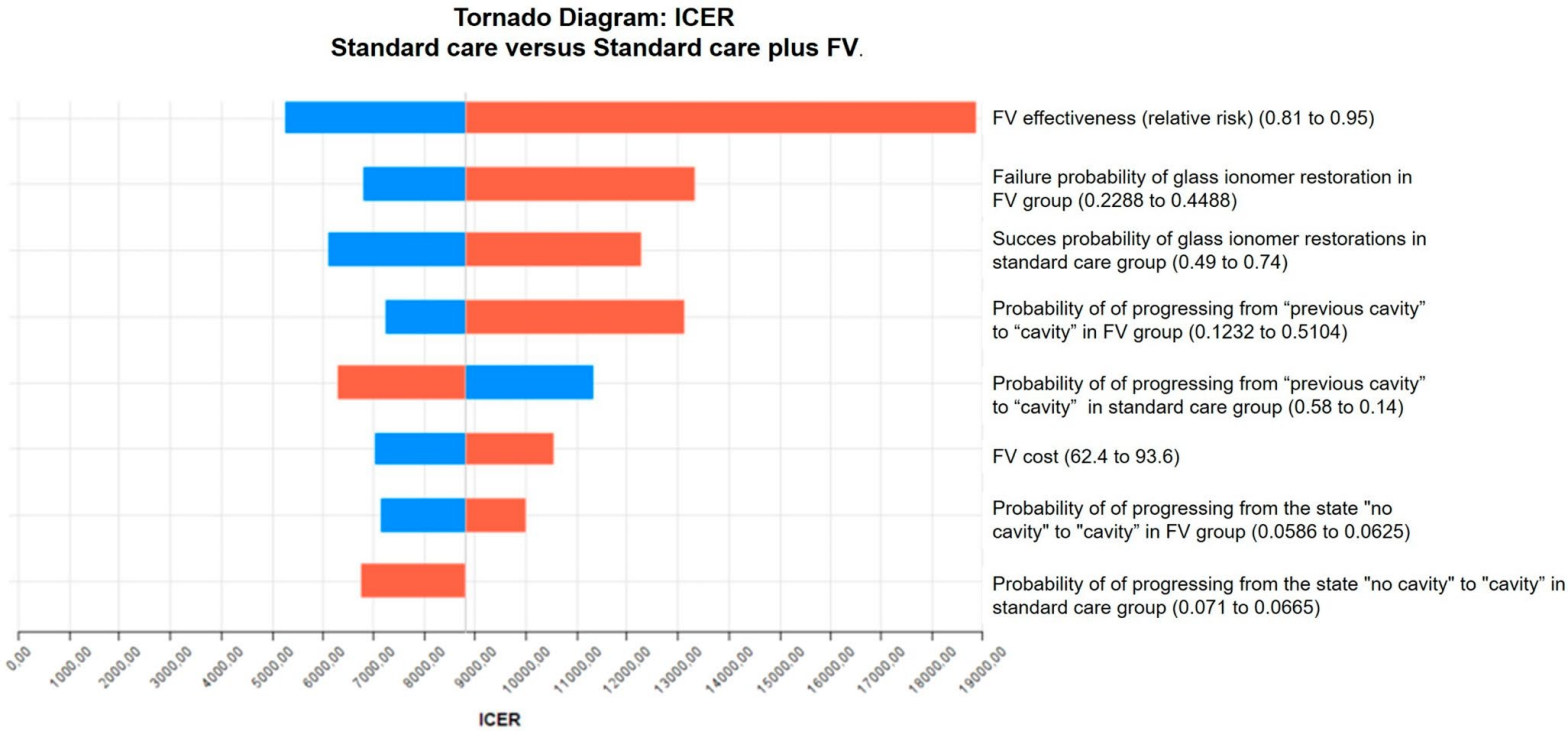
Tornado diagram representing the univariate deterministic sensitivity analyses (cavity) of the cost‐effectiveness analyses of the professional application of fluoride varnish to preschoolers in Brazil, with a 4‐year time horizon and 6‐month cycles. The rectangles represented in orange indicate an increase in the value of the variable. The blue rectangles indicate a decrease in the value of the variable. Thus, when the orange rectangles are on the right side of the image, it means that the increase in the value of a variable results in an increase in the ICER. When the orange rectangles are on the left side of the image, it means that the increase in the value of a variable results in a decrease in the ICER.

**FIGURE 3:**
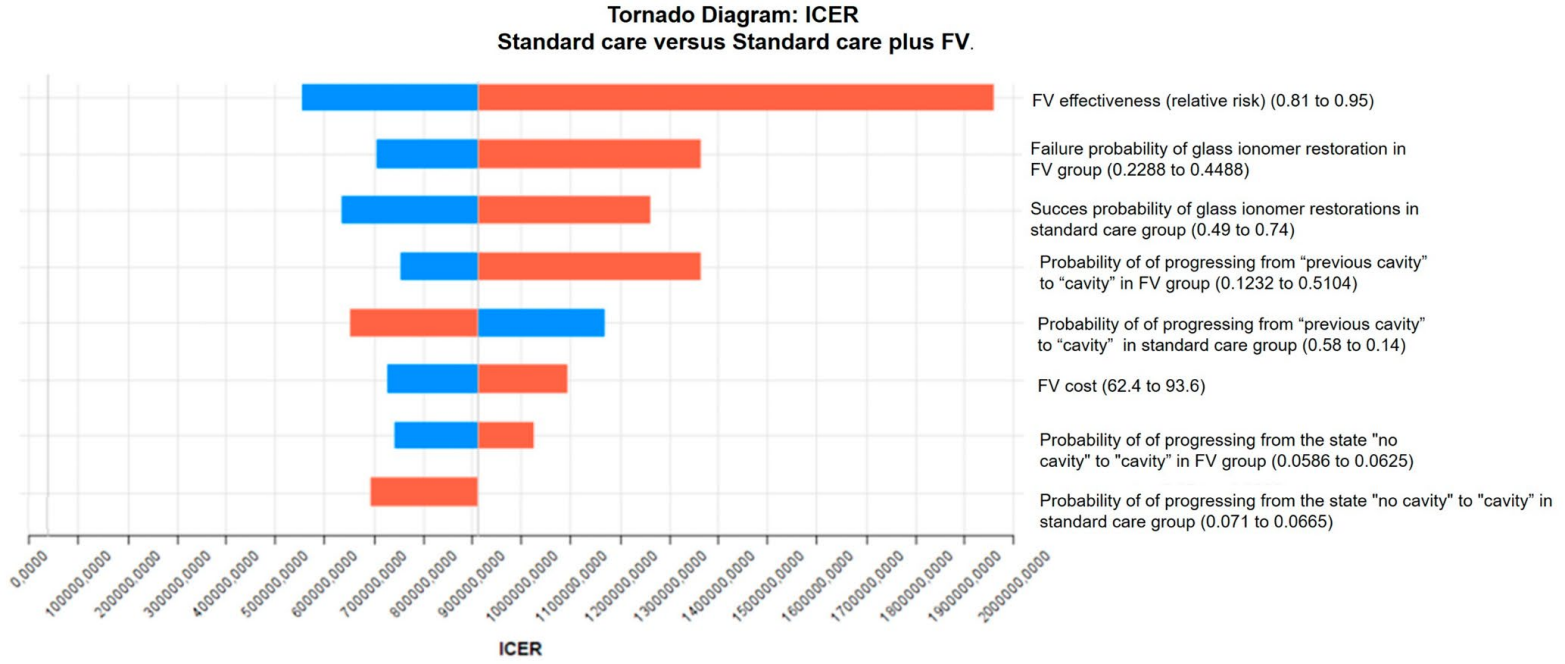
Tornado diagram representing univariate deterministic sensitivity analyses (DALY). The rectangles represented in orange indicate an increase in the value of the variable. The blue rectangles indicate a decrease in the value of the variable. Thus, when the orange rectangles are on the right side of the image, it means that the increase in the value of a variable results in an increase in the ICER. When the orange rectangles are on the left side of the image, it means that the increase in the value of a variable results in a decrease in the ICER.

Results of the univariate deterministic sensitivity analyses are shown in Figures 2 and 3. The results presented in this diagram show that the variable that most affects the ICER measurement is the effectiveness of the FV, inserted into the model in the form of relative risk (“eff_rr”).

**FIGURE 4:**
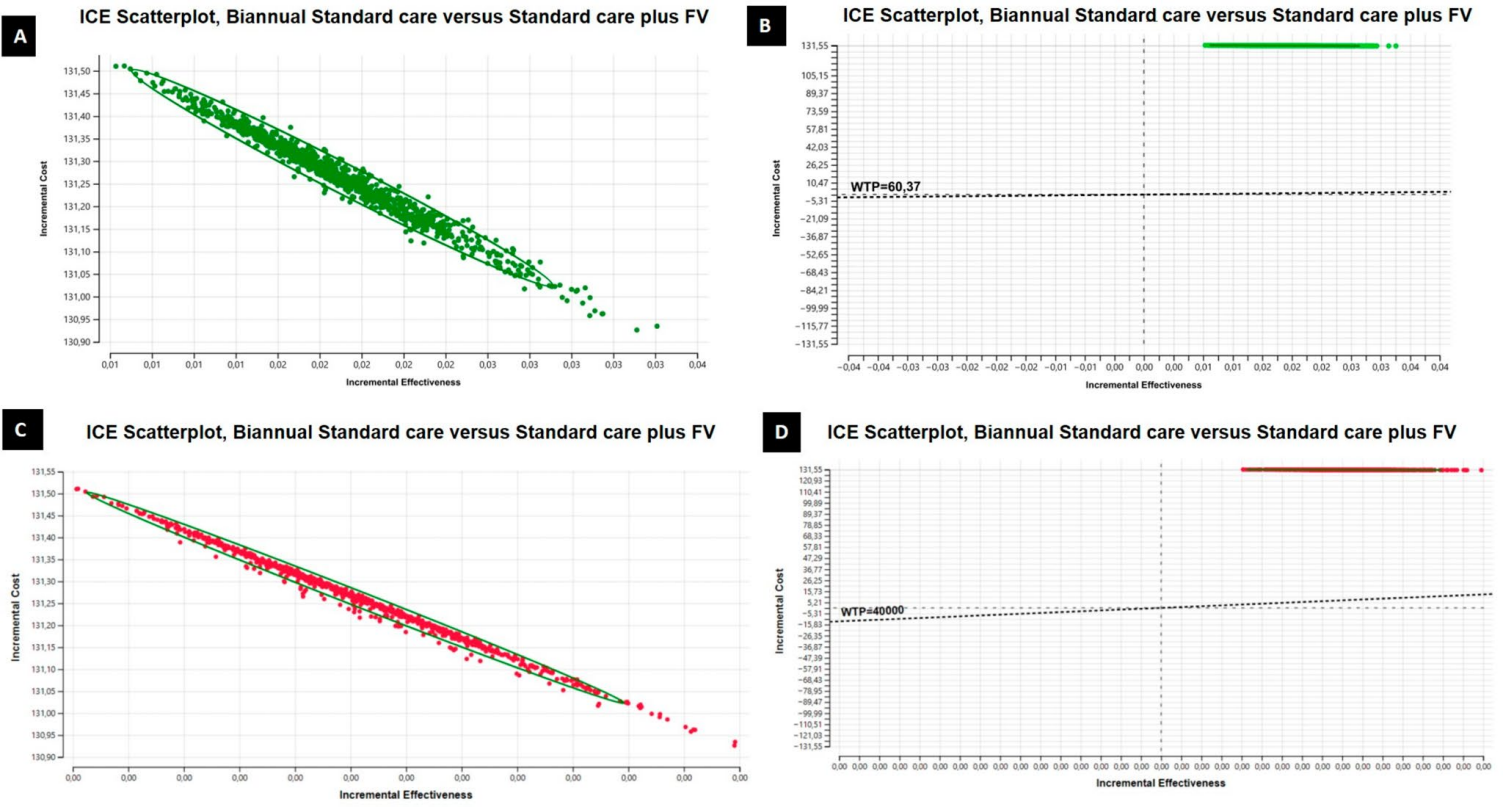
Incremental cost‐effectiveness scatter plots comparing standard care with standard care plus FV. (A) Results for cavity avoided; (B) Results for cavity avoided on a broader scale considering willingness to pay (WTP); (C) Results for DALY avoided; (D) Results for DALY avoided on a broader scale considering willingness to pay (WTP). (A) Results for cavities avoided: Each dot represents a result of cost‐effectiveness in preventing cavities obtained after a simulation. The ellipse highlights the area with the most simulation results, indicating consistent findings. (B) Results for cavities avoided on a broader scale considering willingness to pay (WTP): The dashed line marks the maximum cost society is willing to pay per cavity avoided. Points above this line suggest the intervention may not be cost‐effective. (C) Results for DALYs avoided: Each dot represents a result of cost‐effectiveness in preventing disability‐adjusted life years lost obtained after a simulation, with the ellipse marking the main concentration of results. (D) Results for DALYs avoided on a broader scale considering willingness to pay (WTP): The dashed line shows the WTP threshold per DALY avoided, with points above it indicating the intervention might not be cost‐effective.

The 1000 iterations of the Monte Carlo simulation are displayed in the scatter plots in Figure 4. The results of this sensitivity analysis show no substantial variation in costs, and even less in effectiveness. Each point in the scatter plots represents one simulated outcome comparing incremental costs and effectiveness of standard care versus standard care plus fluoride varnish (FV).

Most of the 1000 iterations simulated are within the ellipse (image 4 A and C). When we evaluate the results plotted in image 4 (B and D), using a broader range of parameters, that is, an exploratory extrapolation of WTP threshold originally defined for cost per QALY to outcomes measured in DALYs or caries lesions averted, we notice that all the results are above the willingness‐to‐pay threshold (WTP) plotted. Points above the dashed WTP line indicate scenarios where the incremental cost per health outcome exceeds the accepted threshold, suggesting the intervention may not be cost‐effective in these cases. If we consider that the QALY‐based threshold can be applied to studies with a DALY outcome (in an approximation), there is no doubt that FV would not be considered cost‐effective.

**FIGURE 5:**
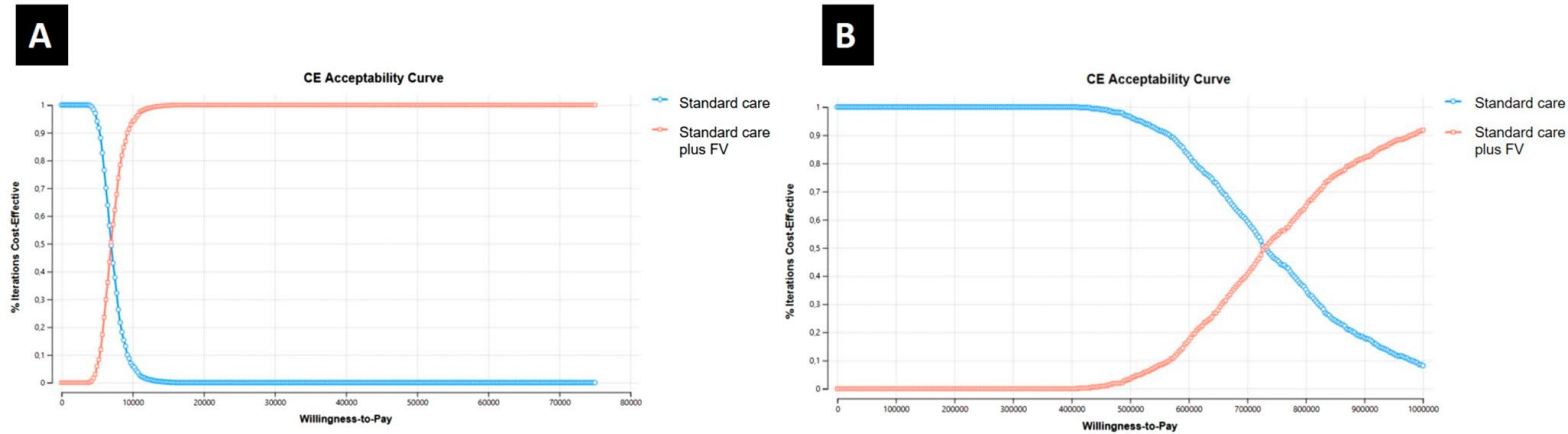
Acceptability curve comparing cost‐effectiveness of standard care versus standard care plus fluoride varnish (A) cavity avoided; (B) DALY avoided.

The acceptability curve from the probabilistic sensitivity analysis is presented in Figure 5. The results indicate that fluoride varnish (FV) would only be considered cost‐effective at relatively high willingness‐to‐pay thresholds: approximately R$7000 to prevent one child from developing a cavitated caries lesion (Figure 5A), and around R$730000 to prevent one DALY (Figure 5B).

## 4 Discussion

The population considered in our analysis consists of preschool children (primary dentition), most likely using fluoride toothpaste. The cost‐effectiveness results based on the two models, for example, considering cavity avoided and DALY avoided, lead us to the following interpretation: Fluoride varnish has a high cost and low effectiveness for the general Brazilian preschool population, considering standard dental care practices, which include not only the cost of dentists applying FV in clinical settings but also the costs associated with restorative and preventive treatments, as well as extractions. The results of the cost‐effectiveness analysis and Markov simulation indicated a cumulative cost of 334 reais per child in the group that received standard care plus FV compared to 203 reais in the standard care group over a period of 4 years, reflecting an additional cost of 131 reais per child, or 33 reais per child per year. This higher cost resulted in an increase in the number of caries‐free children from 57 to 61; thus, FV prevented cavities in 4 out of every 100 children over a 4‐year period, at an average annual cost of R$33 per child (assuming each child who developed cavitated caries lesions had only one). Thus, investing in standard preventive care (oral hygiene and dietary instructions) plus FV is not financially advantageous compared to standard preventive care without FV. To benefit just 4 children, 100 must receive FV, meaning that 96 will receive it without deriving any benefit. Our analysis did not assess whether standard preventive care is financially advantageous compared to no standard preventive care (no intervention). The deterministic sensitivity analyses generally indicated that the effectiveness of the FV is the parameter that most affects the result. The probabilistic analyses revealed that, despite the variability of the results depending on each parameter and the 1000 iterations simulated, there were no significant changes in costs and even fewer in effectiveness.

There is no universally applicable willingness‐to‐pay threshold for the two outcomes assessed in this study. These thresholds, while offering guidance, have limitations. They do not consider the availability of financial resources, nor patient preferences. They generally oversimplify complex decisions. In fact, the Brazilian Ministry of Health only recently suggested a threshold, noting that cost‐effectiveness should support, but not determine, isolated decisions [21]. Other approaches, such as Multicriteria Decision Analysis (MCDA), may complement the assessment, and broader decision‐making criteria must be considered [21, 25, 26].

The relatively high cost of fluoride varnish compared to its modest effectiveness led us to question whether investing in this technology represents the most efficient use of resources allocated for caries control. For instance, it is worth considering whether the distribution of fluoride toothpaste might be a more cost‐effective strategy to ensure that children maintain a daily use of fluoride [12, 24].

The simulation model in this study assumed daily use of fluoride toothpaste by children, an assumption that is generally realistic, given that nearly all toothpastes available in the Brazilian market contain fluoride. However, this assumption may not be valid for all children, as some may lack daily access due to financial constraints. This highlights an important public policy implication: ensuring universal access to fluoride toothpaste may be a more equitable and cost‐effective approach to caries prevention than investing in FV programs.

The results of the present modelling study are in accordance with our synthesis of the evidence on this topic: no convincing overall evidence that applying FV to all preschoolers is a cost‐effective anticaries measure [13].

A mathematical model can be defined as a structure that portrays certain aspects of reality with an adequate degree of detail to support a clinical or political decision [27]. There are different types of models suitable for the same decision problem. Other models could have been proposed for this same problem (and indeed have been in other studies), considering other information, such as additional restorative materials, the number of surfaces affected by caries, and the number of teeth affected by caries simultaneously, among others. This additional information would tend to increase the complexity of the model, and its use would be subject to the information available in the literature.

One limitation of our study was the absence of subgroup analyses, particularly those focusing on high‐risk children. For example, a previous cost‐effectiveness study of FV with a lifetime horizon, conducted with patients aged 6–18 years, concluded that it is unlikely that FV would be cost‐effective in low‐caries‐risk populations [14]. For fluoride varnish to be cost‐effective, material costs would need to be reduced, and its application should take place outside clinical settings and be performed by personnel other than dentists, whose time is costly [14]. Our analysis was conducted at the individual level, acknowledging that each child has 20 primary teeth, all of which could contribute to the simulation outcomes. However, incorporating this level of complexity into the model would have made the analysis more difficult to perform, interpret, and communicate. Nevertheless, our simplified model appears to reasonably reflect real‐world costs and effectiveness. For example, by assuming only one cavitated caries lesion per child, a scenario with four children each having one cavity could, in a more complex model, be equivalent to two children having two cavitated caries lesions each. The resulting differences in cost‐effectiveness estimates between these two scenarios are probably not very large. It is also important to note that, although the model was developed at the individual level, some parameters, such as the probabilities of treatment success and failure, were obtained from studies conducted at the tooth level. This approach was adopted considering the type of data available in the literature and has been acknowledged as a limitation of our study.

Another limitation is the use of epidemiological data from the 2010 National Oral Health Survey. At the time the study was conducted, this was the most recent publicly available national database. We acknowledge that more recent data might have resulted in slightly different estimates. There is no reason to believe, however, that our estimates would have drastically changed since the prevalence of untreated caries in primary teeth has remained stable globally from 1990 to 2021 [28].

We also acknowledge that the effectiveness parameter used in the model, a pooled relative risk (RR) derived from a systematic review and meta‐analysis, was not estimated in a Brazilian population and was generally based on comparisons between FV with usual care or no intervention. However, this RR represents the most updated and comprehensive evidence currently available from high‐quality randomised controlled trials in preschool children. To address potential uncertainty and strengthen the robustness of our model, we incorporated deterministic and probabilistic sensitivity analyses using the confidence intervals reported in the meta‐analysis. This allowed us to explore how variation in the RR could impact cost‐effectiveness outcomes. Nevertheless, given the methodological and data‐related limitations described, our results should be interpreted with caution.

In addition, model validation was conducted through an iterative process involving multiple meetings with paediatric dentistry experts who reviewed and refined the model structure, assumptions, and parameters to ensure face validity. This multidisciplinary approach helped confirm that the model realistically simulates the clinical decision problem and reflects expected outcomes based on existing literature. While external validation with independent data was limited by availability, these steps align with best practices in economic modelling transparency and validation [29], and contribute to the robustness and credibility of our findings.

In the present study, the estimated total cost of care, including the treatment of cavitated caries lesions that were not prevented, was lower in the group that invested less in prevention (without FV) compared to the group that invested more (with FV). While the notion that “more prevention is always better” is widespread, economic evaluations highlight that not all preventive strategies offer good value for money. Future research should explore whether the relatively small number of cavities prevented by fluoride varnish translates into meaningful benefits, such as reductions in dental pain, psychological distress, school absenteeism, and other social consequences associated with caries.

## Author Contributions

Izabel Monteiro Dhyppolito, Rodolfo Castro, Ana Paula Pires dos Santos, and Paulo Nadanovsky contributed to the conception and design of the work as well as the acquisition, analysis, interpretation of data, and drafting the manuscript. All authors critically revised the paper for important intellectual content; gave final approval of the version to be published; and agreed to be accountable for all aspects of the work in ensuring that questions related to the accuracy or integrity of any part of the work are appropriately investigated and resolved.

## Conflicts of Interest

The authors declare no conflicts of interest.

## Supporting information


**Data S1:** Supplementary Appendix.

## Data Availability

The data that support the findings of this study are openly available in OSF.IO at https://osf.io/t6gsx/. DOI: 10.17605/OSF.IO/KC2XN.
