# Supplementary material for: Cost‐Effectiveness of Applying Fluoride Varnish to Preschoolers in a Brazilian Scenario: An Economic Modelling Study
Source: Community Dent Oral Epidemiol. 2025 Nov 20;54(2):191–202. doi: 10.1111/cdoe.70031 (PMC13000991; doi:10.1111/cdoe.70031)
Supplement: Supplementary file 1 — Data S1: Supplementary Appendix. [file CDOE-54-191-s001.docx]

**Appendix I** - Consolidated Health Economic Evaluation Reporting Standards (CHEERS) 2022 Checklist

**
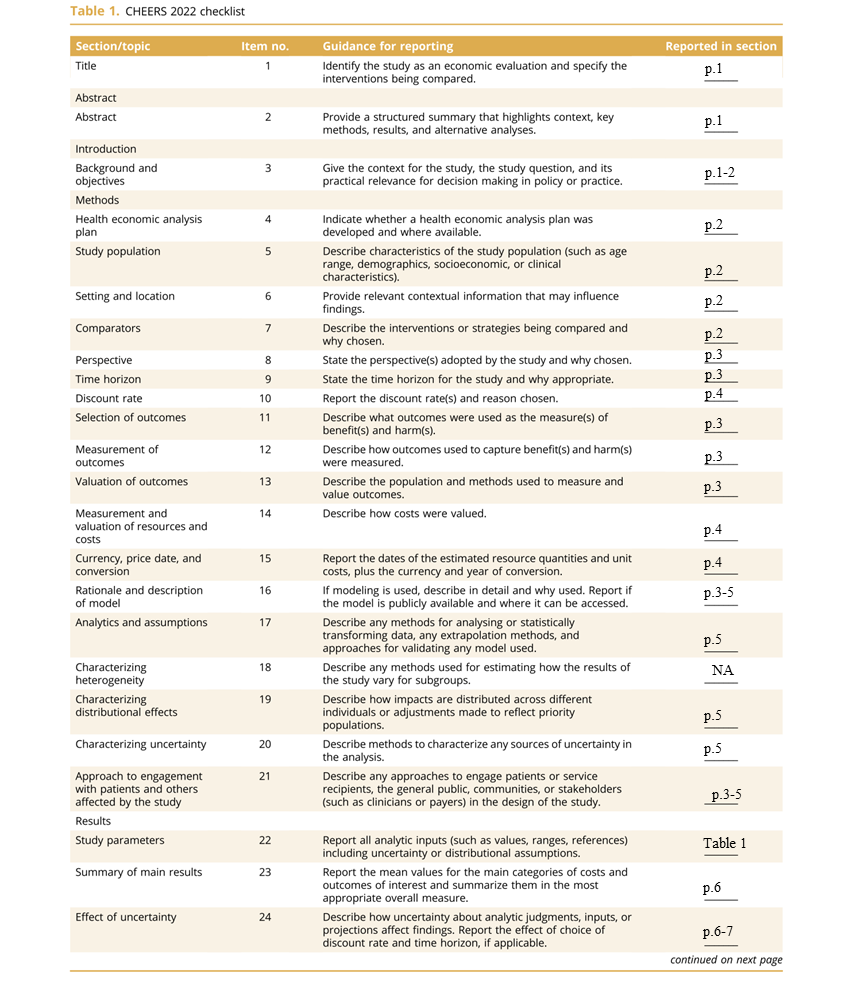
**

**
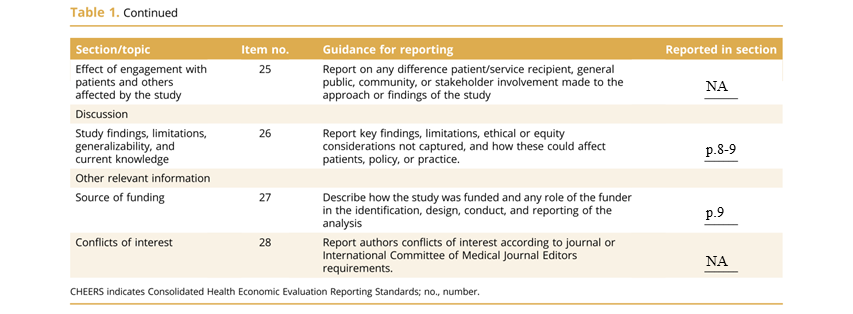
**

**Appendix II** - Markov model tree and probabilities for the cost-effectiveness study of the professional application of fluoride varnish to preschoolers in Brazil

**
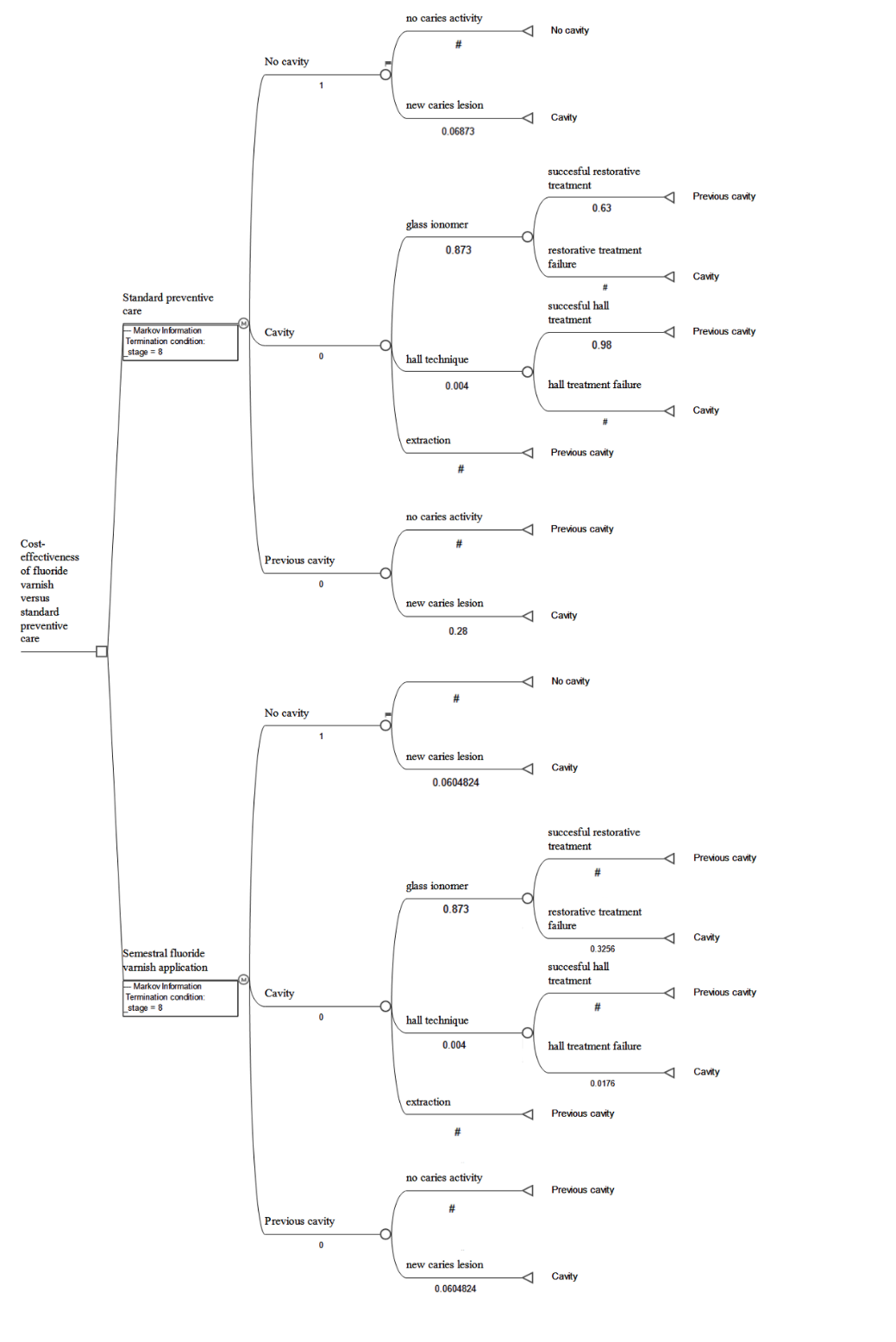
**

This Markov model tree represents the trajectory of individuals over time, with the transition probabilities indicated. All individuals enter the model in a healthy state, that is, free of caries (*no cavity*). After the first cycle, they may either remain in this caries-free state or progress to a state with an active lesion (*cavity*).

Individuals who progress to the *cavity* state necessarily receive treatment, such as a glass ionomer cement restoration, the Hall technique, or an extraction. If the treatment is successful, they move to the *previous cavity* state, which indicates the absence of active lesions but a history of caries.

Alternatively, if the treatment fails, the individual returns to the *cavity* state, meaning the lesion remains active and retreatment is required.

**Appendix III -** ANS price list of procedures, 2022

| **Table code** | **Code TUSS** | **Description TUSS** | **Price** | **INTERVAL TIME (DAYS)** |
| --- | --- | --- | --- | --- |
| **DIAGNÓSTICO (DIAGNOSIS)** | | | | |
| 17 | **81000014** | Condicionamento em Odontologia (Conditioning in Dentistry) | **R$ 56,50** | **4 EVENTS PER 365 DAYS** |
| 17 | **81000049** | Consulta odontológica de Urgência | **R$ 68,00** | **2 EVENTOS A CADA 30 DIAS** |
| 17 | **81000057** | Consulta odontológica de Urgência 24 h | **R$ 92,50** | **_** |
| 17 | **81000065** | Consulta odontológica inicial (Initial dental appointment) | **R$ 38,00** | **180 DAYS** |
| 17 | **81000073** | Consulta odontológica para avaliação técnica de auditoria | **R$ 57,50** | **_** |
| CRITÉRIOS :  Consulta odontológica inicial não requer autorização prévia. Para cada especialidade considera-se o prazo intervalar de 180 dias para cobrança de consultas .  A consulta odontológica de controle corresponde à falta não justificada em tratamentos de longa duração, e somente poderá ser cobrada se o beneficiário assinar um termo de consentimento da cobrança no início do tratamento.  No ano, somente poderão ser cobradas 4 consultas de condicionamento em odontologia, o procedimento destina-se aos beneficiários com até 13 anos incompletos.  As consultas de urgência horário normal são aquelas cujos atendimentos ocorrem das 07:00h às 20:00h. As consultas de urgência 24 h são aquelas cujos atendimentos ocorrem das 20:00h às 07:00h, ou em qualquer horário aos domingos e feriados. SOMENTE PODE SER COBRADA POR CREDENCIADO  CONTRATADO PARA SERVIÇO DE URGÊNCIA 24 HORAS.  Os atendimentos de urgência são cobrados segundo os códigos correspondentes, acrescidos do código dos procedimentos efetivamente realizados. | | | | |
| **RADIOGRAFIAS** | | | | |
| 17 | **81000278** | Fotografia | **R$ 10,00** | **18 EVENTOS A CADA 365 DIAS** |
| 17 | **81000294** | Levantamento Radiográfico (Exame Radiodôntico) | **R$ 128,50** | **2 EVENTOS A CADA 365 DIAS** |
| 17 | **81000308** | Modelos ortodônticos | **R$ 27,50** | **365 DIAS = 1 ANO** |
| 17 | **81000324** | Radiografia antero-posterior | **R$ 49,50** | **365 DIAS = 1 ANO** |
| 17 | **81000340** | Radiografia da ATM | **R$ 107,50** | **_** |
| 17 | **81000367** | Radiografia da mão e punho - carpal | **R$ 49,50** | **_** |
| 17 | **81000375** | Radiografia interproximal - bite-wing | **R$ 12,50** | **4 EVENTOS A CADA 365 DIAS** |
| 17 | **81000383** | Radiografia oclusal | **R$ 30,00** | **2 EVENTOS A CADA 365 DIAS** |
| 17 | **81000405** | Radiografia panorâmica de mandíbula/maxila  (ortopantomografia) | **R$ 63,00** | **2 EVENTOS A CADA 365 DIAS** |
| 17 | **81000413** | Radiografia panorâmica de mandíbula/maxila  (ortopantomografia) com traçado cefalométrico | **R$ 73,00** | **_** |
| 17 | **81000421** | Radiografia periapical | **R$ 12,50** | **10 EVENTOS A CADA 365 DIAS** |
| 17 | **81000456** | Slide | **R$ 11,50** | **_** |
| 17 | **81000472** | Telerradiografia | **R$ 61,00** | **365 DIAS = 1 ANO** |
| 17 | **81000480** | Telerradiografia com traçado cefalométrico | **R$ 78,50** | **2 EVENTOS A CADA 365 DIAS** |
| 17 | **81000510** | Tomografia computadorizada por feixe cônico – cone beam | **R$ 283,00** | **VERIFICAR CRITÉRIO** |
| 17 | **81000529** | Tomografia convencional – linear ou multi-direcional | **R$ 488,00** | **VERIFICAR CRITÉRIO** |
| 17 | **81000537** | Traçado Cefalométrico | **R$ 74,00** | **3 EVENTOS A CADA 365 DIAS** |
| 00 | **89130124** | Modelo de Estudo (ORTODONTIA-ZOCALADOS) -  Superior e Inferior (*) | **R$ 80,50** | **2 EVENTOS A CADA 365 DIAS** |

Note: To preserve the original context, the full table is provided in Portuguese. Only the data relevant to this study were translated into English.

| **Table code** | **CÓD. TUSS** | **Descrição TUSS** | **Valor** | **PRAZO INTERVALAR (DIAS)** |
| --- | --- | --- | --- | --- |
| CRITÉRIOS :  SOMENTE É NECESSÁRIA AUTORIZAÇÃO PRÉVIA PARA TOMOGRAFIA.  Os limites para cobrança são os abaixo descritos:   - Radiografia Panorâmica: 01 a cada 365 dias. - Radiografia Bite wings: 04 a cada 365 dias - Radiografia oclusal: 02 a cada 365 dias - Radiografia periapical: de acordo com os procedimentos necessários. - Fotografia: 08 a cada 365 dias. - Levantamento Radiográfico: 01 a cada 365 dias. - Modelos Ortodônticos: 02 a cada 365 dias.- - Tomografia Cone Bean: 02 exames de ARCADA, 02 (dois) exames de SEGMENTO ou 01 (um) exame contendo ARCADA E SEGMENTO a cada 365 dias   Na necessidade de realização dos procedimentos acima em prazo intervalar inferior ao descrito, deve ser enviado à FIOSAÚDE relatório técnico com justificativa junto à cobrança.  (*) Os modelos de estudo (ortodontia zocalados) somente poderão ser realizados por prestadores que possuam credenciamento na especialidade Radiologia odontológica.   - Fotografia: 08 a cada 365 dias. | | | | |
| **PREVENÇÃO (PREVENTING)** | | | | |
| 17 | **84000031** | Aplicação de cariostático (Cariostatic application) | **R$ 21,50** | **365 DAYS = 1 YEAR** |
| 17 | **84000074** | Aplicação de selante de fóssulas e fissuras (Fissure sealant) | **R$ 39,00** | **180 DAYS** |
| 17 | **84000090** | Aplicação tópica de fluor (por arcada) (Topical fluoride application (per arch)) | **R$ 19,50** | **CHECK CRITERIA** |
| 17 | **84000139** | Atividade educativa em saúde bucal (Oral health education) | **R$ 28,00** | **180 DAYS** |
| 17 | **84000163** | Controle de biofilme (placa bacteriana) (Biofilm (plaque) control) | **R$ 25,50** | **180 DAYS** |
| 17 | **84000198** | Profilaxia: polimento coronário (Dental Prophylaxis) | **R$ 27,00** | **365 DAYS = 1 YEAR** |
| 17 | **84000201** | Remineralização (Remineralization) | **R$ 29,00** | **180 DAYS** |
| 17 | **85300047** | Raspagem supra-gengival ( por arcada )(Supragingival scraping (per arch)) | **R$ 86,50** | **365 DAYS = 1 YEAR** |
| 00 | **89120102** | Avaliação do risco de cárie (Caries risk assessment) | **R$ 46,50** | **365 DAYS = 1 YEAR** |
| Criteria:The 360-day interval must be observed for supra-gingival scraping, fluoride therapy, cariostatic application, caries risk assessment, prophylaxis and coronal polishing. In special cases, authorization may be granted to reduce the interval period, upon submission of a technical justification for evaluation by FIOSAÚDE.The 180-day interval must be observed for enamel remineralization procedures, plaque evidence and educational activities.Supragingival scraping or prophylaxis cannot be carried out concurrently with subgingival scraping.  Sub-gingival scraping cannot be carried out concurrently with periodontal surgery, topical fluoride application, prophylaxis, polishing and other preventive measures.The 180-day interval must be observed in the sealant application procedure. The procedure can only be carried out on patients between the ages of 6 and 14 who have eruption and a risk of caries. This procedure already includes prophylaxis. Sealants cannot be applied over restorations. The face of the element on which the sealant will be applied must be informed.Remineralization will be according to the patient's caries risk. Only up to 4 sessions every 90 days can be charged. The caries risk assessment includes a salivary flow test. | | | | |
| **ODONTOPEDIATRIA (PEDIATRIC DENTISTRY)** | | | | |
| 17 | **83000046** | Coroa de aço em dente decíduo (Stainledd steel crown restorations) | **R$ 136,00** | **365 DIAS = 1 ANO** |
| 17 | **83000062** | Coroa de policarbonato em dente decíduo | **R$ 136,00** | **_** |
| 17 | **83000089** | Exodontia simples de decíduo (Deciduous extraction) | **R$ 54,50** | **_** |
| 17 | **83000097** | Mantenedor de espaço fixo | **R$ 223,00** | **730 DIAS = 2 ANOS** |
| 17 | **83000100** | Mantenedor de espaço removível | **R$ 223,00** | **365 DIAS = 1 ANO** |
| 17 | **83000127** | Pulpotomia em dente decíduo | **R$ 81,50** | **_** |
| 17 | **83000151** | Tratamento endodôntico em dente decíduo | **R$ 127,50** | **_** |
| 17 | **84000163** | Controle de biofilme (placa bacteriana)(Biofilm (plaque) control) | **R$ 25,50** | **_** |
| 17 | **85100013** | Capeamento pulpar direto (*) (Direct pulp capping (*)) | **R$ 69,50** | **2 EVENTOS A CADA 365 DIAS** |

| **Table code** | **CÓD. TUSS** | **Descrição TUSS** | **Valor** | **PRAZO INTERVALAR (DIAS)** |
| --- | --- | --- | --- | --- |
| CRITÉRIOS :  Todos os procedimentos desta especialidade requerem autorização prévia, exceto exodontia de decíduo, capeamento pulpar direto e pulpotomia.  Os tratamentos em odontopediatria destinam-se aos beneficiários com até 13 anos incompletos.  No ano, somente poderão ser cobradas 4 consultas de condicionamento em odontologia - especialidade - Diagnóstico - código - 81000014. | | | | |
| **DENTÍSTICA (RESTORATIVE DENTISTRY)** | | | | |
| 17 | **85100013** | Capeamento pulpar direto (*) | **R$ 69,50** | **2 EVENTOS A CADA 365 DIAS** |
| 17 | **85100048** | Colagem de fragmentos dentários | **R$ 128,50** | **_** |
| 17 | **85100064** | Faceta direta em resina fotopolimerizável | **R$ 121,50** | **_** |
| 17 | **85100099** | Restauração de amálgama - 1 face (*) | **R$ 61,00** | **365 DIAS = 1 ANO** |
| 17 | **85100102** | Restauração de amálgama - 2 faces (*) | **R$ 74,00** | **365 DIAS = 1 ANO** |
| 17 | **85100110** | Restauração de amálgama - 3 faces (*) | **R$ 89,00** | **365 DIAS = 1 ANO** |
| 17 | **85100129** | Restauração de amálgama - 4 faces (*) | **R$ 115,50** | **365 DIAS = 1 ANO** |
| 17 | **85100137** | Restauração em ionômero de vidro - 1 face (*)(Glass ionomer restoration – 1 surface) | **R$ 61,00** | **365 DIAS = 1 ANO** |
| 17 | **85100145** | Restauração em ionômero de vidro - 2 faces (*)(Glass ionomer restoration – 2 surfaces) | **R$ 61,00** | **365 DIAS = 1 ANO** |
| 17 | **85100153** | Restauração em ionômero de vidro - 3 faces (*)(Glass ionomer restoration – 3 surfaces) | **R$ 61,00** | **365 DIAS = 1 ANO** |
| 17 | **85100161** | Restauração em ionômero de vidro - 4 faces (*) (Glass ionomer restoration – 4 surfaces) | **R$ 61,00** | **365 DIAS = 1 ANO** |
| 17 | **85100196** | Restauração em resina fotopolimerizável 1 face (*) | **R$ 69,50** | **365 DIAS = 1 ANO** |
| 17 | **85100200** | Restauração em resina fotopolimerizável 2 faces (*) | **R$ 81,50** | **365 DIAS = 1 ANO** |
| 17 | **85100218** | Restauração em resina fotopolimerizável 3 faces (*) | **R$ 95,50** | **365 DIAS = 1 ANO** |
| 17 | **85100226** | Restauração em resina fotopolimerizável 4 faces (*) | **R$ 103,00** | **365 DIAS = 1 ANO** |
| 17 | **85200085** | Restauração temporária / tratamento expectante (*) | **R$ 33,50** | **_** |
| 17 | **85300012** | Dessensibilização dentária | **R$ 33,50** | **180 DIAS** |
| 17 | **85400262** | Pino Pré Fabricado (*) | **R$ 26,00** | **2 EVENTOS A CADA 365 DIAS** |
| CRITÉRIOS :  Os procedimentos com (*) não necessitam de autorização prévia. Observar os critérios abaixo.  Os profissionais e clínicas credenciados devem controlar, em prontuário clínico, a execução de todos os procedimentos, respeitando o prazo intervalar, desde a consulta inicial, buscando informações junto ao paciente das datas de realização de tratamentos. Os prazos deverão estar em conformidade com a lista a seguir:   - Restaurações fotopolimerizáveis, amálgamas, restaurações ionômero: o prazo é de 365 dias. - Capeamento pulpar: 02 a cada 365 dias.   Na necessidade de realização dos procedimentos acima em prazo intervalar inferior ao descrito, deve ser enviado à Fiosaúde relatório técnico com justificativa para o e-mail [odontologia@fiosaude.org.br.](mailto:odontologia@fiosaude.org.br)  Os procedimentos de colagem de fragmentos, faceta direta em resina fotopolimerizável e dessensibilização dentária necessitam de autorização prévia.  O procedimento de dessensibilização dentária será pago por dente, independente de técnicas e ou número de sessões realizadas e possui prazo intervalar de 180 dias. | | | | |
| **ENDODONTIA** | | | | |
| 17 | **82000077** | Apicetomia birradiculares com obturação retrógrada | **R$ 292,00** | **_** |
| 17 | **82000158** | Apicetomia multirradiculares com obturação retrógrada | **R$ 305,00** | **_** |
| 17 | **82000174** | Apicetomia unirradiculares com obturação retrógrada | **R$ 239,50** | **_** |
| 17 | **85100056** | Curativo de demora em endodontia | **R$ 125,50** | **_** |
| 17 | **85200018** | Clareamento de dente desvitalizado | **R$ 139,00** | **_** |
| 17 | **85200034** | Pulpectomia | **R$ 72,00** | **_** |
| 17 | **85200042** | Pulpotomia | **R$ 72,00** | **_** |
| 17 | **85200077** | Remoção de núcleo intrarradicular | **R$ 103,50** | **_** |
| 17 | **85200093** | Retratamento endodôntico birradicular | **R$ 334,50** | **_** |
| 17 | **85200107** | Retratamento endodôntico multirradicular | **R$ 459,00** | **_** |
| 17 | **85200115** | Retratamento endodôntico unirradicular | **R$ 237,00** | **_** |
| 17 | **85200123** | Tratamento de perfuração endodôntica | **R$ 133,50** | **_** |

| **Table code** | **CÓD. TUSS** | **Descrição TUSS** | **Valor** | **PRAZO INTERVALAR (DIAS)** |
| --- | --- | --- | --- | --- |
| 17 | **85200140** | Tratamento endodôntico birradicular | **R$ 306,00** | **_** |
| 17 | **85200158** | Tratamento endodôntico multirradicular | **R$ 389,00** | **_** |
| 17 | **85200026** | Preparo Intra Canal | **R$ 180,50** | **_** |
| 17 | **85200166** | Tratamento endodôntico unirradicular | **R$ 144,50** | **_** |
| CRITÉRIOS :  Todos os procedimentos desta especialidade requerem autorização prévia.  As solicitações de retratamento e os tratamentos endodônticos com finalidade protética serão encaminhadas à auditoria documental. As radiografias iniciais devem ser enviadas através do email [odontologia@fiosaude.org.br.](mailto:odontologia@fiosaude.org.br)  Ao final do tratamento, a radiografia final deve ser encaminhada para auditoria documental final através do email [odontologia@fiosaude.org.br.](mailto:odontologia@fiosaude.org.br) | | | | |
| **PERIODONTIA** | | | | |
| 17 | **82000212** | Aumento de coroa clínica | **R$ 166,50** | **_** |
| 17 | **82000417** | Cirurgia periodontal a retalho | **R$ 154,00** | **_** |
| 17 | **82000557** | Cunha proximal | **R$ 121,50** | **_** |
| 17 | **82000581** | Enxerto com osso autógeno da linha oblíqua | **R$ 436,50** | **_** |
| 17 | **82000603** | Enxerto com osso autógeno do mento | **R$ 436,50** | **_** |
| 17 | **82000620** | Enxerto com osso liofilizado | **R$ 366,00** | **_** |
| 17 | **82000646** | Enxerto conjuntivo subepitelial | **R$ 204,50** | **_** |
| 17 | **82000689** | Enxerto pediculado | **R$ 187,00** | **_** |
| 17 | **82000921** | Gengivectomia | **R$ 154,50** | **_** |
| 17 | **82000948** | Gengivoplastia | **R$ 154,50** | **_** |
| 17 | **82001073** | Odonto-secção | **R$ 129,50** | **_** |
| 17 | **82001464** | Sepultamento radicular | **R$ 140,00** | **_** |
| 17 | **82001707** | Ulectomia | **R$ 120,50** | **_** |
| 17 | **82001715** | Ulotomia | **R$ 120,50** | **_** |
| 17 | **85300020** | Imobilização dentária em dentes permanentes | **R$ 46,50** | **_** |
| 17 | **85300039** | Raspagem sub-gengival/alisamento radicular | **R$ 19,00** | **180 DIAS** |
| 17 | **85300063** | Tratamento de abscesso periodontal agudo | **R$ 80,50** | **_** |
| 17 | **89140235** | Raspagem Sub Gengival por sexteto (*) | **R$ 90,50** | **180 DIAS** |
| 17 | **89140240** | Consulta clínica - Planejamento de tratamento periodontal | **R$ 56,50** | **180 DIAS** |
| CRITÉRIOS :  Todos os procedimentos desta especialidade requerem autorização prévia.  (*) Na raspagem sub gengival por sexteto, o segmento será cobrado quando estiverem presentes no mínimo 03 elementos.  O prazo intervalar de 180 dias deve ser observado nos procedimentos de raspagem sub gengival, que não pode ser realizado concomitantemente com a cirurgia Periodontal. A raspagem subgengival também não pode ser realizada ao mesmo tempo que raspagem supra, aplicação tópica de flúor, profilaxia, polimento  e outros itens de prevenção. | | | | |
| **PRÓTESE DENTAL** | | | | |
| 17 | **81000243** | Diagnóstico por meio de enceramento | **R$ 45,50** | **_** |
| 17 | **81000260** | Diagnóstico por meio de procedimentos laboratoriais | **R$ 127,50** | **_** |
| 17 | **85067110** | Coroa Veneer | **R$ 448,00** | **_** |
| 17 | **85067212** | Prótese Parcial Removível com estrutura metálica unilateral | **R$ 590,50** | **_** |
| 17 | **85067256** | Encaixe (fêmea) por elemento | **R$ 389,50** | **_** |
| 17 | **85400025** | Ajuste Oclusal por desgaste seletivo | **R$ 57,50** | **_** |
| 17 | **85400068** | Conserto em prótese total (exclusivamente em consultório) | **R$ 101,00** | **_** |
| 17 | **85400076** | Coroa provisória com pino | **R$ 79,50** | **365 DIAS = 1 ANO** |
| 17 | **85400084** | Coroa provisória sem pino | **R$ 79,50** | **365 DIAS = 1 ANO** |
| 17 | **85400092** | Coroa total acrílica prensada | **R$ 200,50** | **365 DIAS = 1 ANO** |

| **Table code** | **CÓD. TUSS** | **Descrição TUSS** | **Valor** | **PRAZO INTERVALAR (DIAS)** |
| --- | --- | --- | --- | --- |
| 17 | **85400106** | Coroa total em cerâmica pura | **R$ 788,00** | **1040 DIAS** |
| 17 | **85400114** | Coroa total em cerômero | **R$ 401,00** | **730 DIAS = 2 ANOS** |
| 17 | **85400149** | Coroa total metálica | **R$ 467,50** | **730 DIAS = 2 ANOS** |
| 17 | **85400157** | Coroa total metalo cerâmica | **R$ 788,00** | **1080 DIAS** |
| 17 | **85400181** | Faceta em cerâmica pura | **R$ 803,50** | **1080 DIAS** |
| 17 | **85400203** | Guia cirúrgico para prótese total imediata | **R$ 194,50** | **_** |
| 17 | **85400211** | Núcleo de preenchimento | **R$ 83,50** | **365 DIAS = 1 ANO** |
| 17 | **85400220** | Núcleo metálico fundido | **R$ 158,50** | **730 DIAS = 2 ANOS** |
| 17 | **85200077** | Remoção de núcleo intra radicular | **R$ 103,50** | **_** |
| 17 | **85400270** | Placa Ocusal resiliente | **R$ 238,50** | **VERIFICAR CRITÉRIO** |
| 17 | **85400246** | Órtese miorrelaxante (placa oclusal estabilizadora) | **R$ 551,00** | **730 DIAS = 2 ANOS** |
| 17 | **85400289** | Prótese fixa adesiva direta (provisória) | **R$ 261,00** | **_** |
| 17 | **85400300** | Prótese fixa adesiva indireta em metalo cerâmica | **R$ 1.165,00** | **_** |
| 17 | **85400319** | Prótese fixa adesiva indireta em metalo plástica | **R$ 802,50** | **_** |
| 17 | **85400335** | Prótese parcial fixa em metalo cerâmica | **R$ 830,00** | **1095 DIAS = 3 ANOS** |
| 17 | **85400343** | Prótese parcial fixa em metalo plástica | **R$ 517,50** | **1095 DIAS = 3 ANOS** |
| 17 | **85400360** | Prótese parcial fixa provisória | **R$ 80,50** | **730 DIAS = 2 ANOS** |
| 17 | **85400378** | Prótese parcial removível com encaixes de precisão ou de  semi precisão | **R$ 1.271,00** | **1095 DIAS = 3 ANOS** |
| 17 | **85400386** | Prótese parcial removível com grampos bilateral | **R$ 1.002,50** | **1095 DIAS = 3 ANOS** |
| 17 | **85400394** | Prótese parcial removível provisória em acrílico com ou sem  grampos | **R$ 479,50** | **1095 DIAS = 3 ANOS** |
| 17 | **85400408** | Prótese total | **R$ 1.137,50** | **1095 DIAS = 3 ANOS** |
| 17 | **85400416** | Prótese total imediata | **R$ 802,50** | **_** |
| 17 | **85400424** | Prótese total incolor | **R$ 1.137,50** | **1095 DIAS = 3 ANOS** |
| 17 | **85400467** | Recimentação de trabalhos protéticos | **R$ 52,00** | **60 DIAS** |
| 17 | **85400475** | Reembasamento de coroa provisória | **R$ 33,50** | **_** |
| 17 | **85400483** | Reembasamento de prótese total ou parcial - imediato (em  consultório) | **R$ 219,00** | **365 DIAS = 1 ANO** |
| 17 | **85400505** | Remoção de trabalho protético | **R$ 43,50** | **365 DIAS = 1 ANO** |
| 17 | **85400513** | Restauração em cerâmica pura - inlay | **R$ 599,50** | **1095 DIAS = 3 ANOS** |
| 17 | **85400521** | Restauração em cerâmica pura - onlay | **R$ 599,50** | **1095 DIAS = 3 ANOS** |
| 17 | **85400530** | Restauração em cerômero - onlay | **R$ 456,50** | **1095 DIAS = 3 ANOS** |
| 17 | **85400548** | Restauração em cerômero - inlay | **R$ 456,50** | **1095 DIAS = 3 ANOS** |
| 17 | **85400556** | Restauração metálica fundida | **R$ 346,50** | **730 DIAS = 2 ANOS** |
| 17 | **85500011** | Coroa provisória sobre implante | **R$ 80,50** | **1095 DIAS = 3 ANOS** |
| 17 | **85500038** | Coroa total metalo cerâmica sobre implante | **R$ 830,00** | **_** |
| 17 | **85500089** | Manutenção de prótese sobre implantes | **R$ 117,00** | **_** |
| 17 | **85500097** | Overdenture barra clipe ou o'ring sobre dois implantes | **R$ 1.874,00** | **1095 DIAS = 3 ANOS** |
| 17 | **85500100** | Overdenture barra clipe ou o'ring sobre quatro ou mais  implantes | **R$ 2.132,50** | **1095 DIAS = 3 ANOS** |
| 17 | **85500119** | Overdenture barra clipe ou o'ring sobre três implantes | **R$ 2.007,00** | **_** |
| 17 | **85500160** | Protocolo Branemarj para 04 implantes inferiores | **R$ 2.621,00** | **_** |
| 17 | **85500178** | Protocolo Branemarj para 05 implantes superiores | **R$ 2.621,00** | **_** |
| 17 | **85500186** | Protocolo Branemarj provisório para 04 ou mais implantes  inferiores e ou superiores- por elemento | **R$ 80,50** | **_** |
| 17 | **86000446** | Pistas indiretas de Planas | **R$ 538,50** | **_** |
| 17 | **86000551** | Plano inclinado | **R$ 84,50** | **_** |
| 17 | **89170363** | Modelo de estudo superior e inferior | **R$ 73,00** | **_** |
| 00 | **89170460** | Prótese Total revestida por Silicone | **R$ 1.272,00** | **1095 DIAS = 3 ANOS** |

| **Table code** | **CÓD. TUSS** | **Descrição TUSS** | **Valor** | **PRAZO INTERVALAR (DIAS)** |
| --- | --- | --- | --- | --- |
| CRITÉRIOS :  Todos os procedimentos desta especialidade requerem autorização prévia, à exceção de conserto de prótese, recimentação de trabalhos protéticos, manutenção de próteses , reembasamento de coroa provisória.  O planejamento do tratamento protético é de responsabilidade do prestador, que deverá levar em consideração possíveis hábitos parafuncionais existentes. Não poderá ser imputada ao paciente qualquer responsabilidade após a conclusão do tratamento.  Podem ser solicitados até três ajustes oclusais por tratamento de reabilitação oral.  Para efetuar o pagamento dos procedimentos: placa de mordida, jig ou front platô, a FIOSAÚDE poderá solicitar a apresentação das referidas peças na auditoria.  (*) Para utilização de metais nobres, o prestador deverá informar a liga e a quantidade utilizada para que seja incluído na autorização o valor correspondente ao peso, conforme parâmentros definidos pela FIOSAÚDE.  Admite-se para restauração de núcleo metálico fundido até 3 gramas em incisivos e caninos, até 5g em pré molares e até 6g em molares. Para RMF e Coroas Totais, até 6g.  Para o procedimento de manutenção de próteses sobre implante deve ser observado o prazo intervalar de 365 dias, e a cobrança poderá ser por arcada, mediante a apresentação de relatório técnico justiticativo  A Ortese miorelaxante somente poderá ser realizada por profissionais especializados em Ortodontia, OFM,DTM, Periodontia, Prótese e  Reabilitação Oral, deve ser enviado a justificativa técnica . Compreende a placa encerada e acrilizada. 85400246  A Placa Oclusal resiliente é a realizada em silicone em qualquer gramatura, podendo ser solicitada por qualquer especialidade, e deve ser enviada também a justificativa técnica. 85400270 | | | | |
| **CIRURGIA ORAL MENOR** | | | | |
| 17 | **82000034** | Alveoloplastia | **R$ 120,50** | **_** |
| 17 | **82000085** | Apicetomia birradiculares sem obturação retrógrada | **R$ 281,00** | **_** |
| 17 | **82000166** | Apicetomia multirradiculares sem obturação retrógrada | **R$ 294,00** | **_** |
| 17 | **82000182** | Apicetomia unirradiculares sem obturação retrógrada | **R$ 227,50** | **_** |
| 17 | **82000190** | Aprofundamento/aumento de vestíbulo | **R$ 128,50** | **_** |
| 17 | **82000239** | Biópsia de boca | **R$ 100,00** | **_** |
| 17 | **82000247** | Biópsia de glândula salivar incisional | **R$ 100,00** | **_** |
| 17 | **82000255** | Biópsia de lábio | **R$ 100,00** | **_** |
| 17 | **82000263** | Biópsia de língua | **R$ 100,00** | **_** |
| 17 | **82000271** | Biópsia de mandíbula | **R$ 100,00** | **_** |
| 17 | **82000280** | Biópsia de maxila | **R$ 100,00** | **_** |
| 17 | **82000298** | Bridectomia | **R$ 140,50** | **_** |
| 17 | **82000360** | Cirurgia para torus mandibular – bilateral | **R$ 202,50** | **_** |
| 17 | **82000387** | Cirurgia para torus mandibular – unilateral | **R$ 132,50** | **_** |
| 17 | **82000395** | Cirurgia para torus palatino | **R$ 132,50** | **_** |
| 17 | **82000468** | Controle de hemorragia com aplicação de agente  hemostático em região buco-maxilo-facial | **R$ 93,50** | **_** |
| 17 | **82000484** | Controle de hemorragia sem aplicação de agente hemostático  em região buco-maxilo-facial | **R$ 79,50** | **_** |
| 17 | **82000778** | Exérese ou excisão de cálculo salivar | **R$ 169,50** | **_** |
| 17 | **82000786** | Exérese ou excisão de cistos odontológicos | **R$ 247,00** | **_** |
| 17 | **82000794** | Exérese ou excisão de mucocele | **R$ 134,00** | **_** |
| 17 | **82000808** | Exérese ou excisão de rânula | **R$ 403,00** | **_** |

| **Table code** | **CÓD. TUSS** | **Descrição TUSS** | **Valor** | **PRAZO INTERVALAR (DIAS)** |
| --- | --- | --- | --- | --- |
| 17 | **82000816** | Exodontia a retalho | **R$ 94,50** | **_** |
| 17 | **82000859** | Exodontia de raiz residual | **R$ 73,00** | **_** |
| 17 | **82000875** | Exodontia simples de permanente | **R$ 76,00** | **_** |
| 17 | **82000883** | Frenulectomia labial | **R$ 115,50** | **_** |
| 17 | **82000891** | Frenulectomia lingual | **R$ 126,50** | **_** |
| 17 | **82000964** | Implante ortodôntico | **R$ 471,50** | **_** |
| 17 | **82001030** | Incisão e Drenagem intra-oral de abscesso, hematoma e/ou  flegmão da região buco-maxilo-facial | **R$ 93,50** | **_** |
| 17 | **82001154** | Reconstrução do sulco gengivo labial | **R$ 129,50** | **_** |
| 17 | **82001197** | Redução simples de luxação de Articulação Têmporo-  mandibular (ATM) | **R$ 158,50** | **_** |
| 17 | **82001243** | Regeneração Tecidual Guiada- RTG | **R$ 315,50** | **_** |
| 17 | **82001251** | Reimplante dentário com contenção | **R$ 141,00** | **_** |
| 17 | **82001286** | Remoção de dentes inclusos / impactados | **R$ 222,00** | **_** |
| 17 | **82001294** | Remoção de dentes semi-inclusos / impactados | **R$ 222,00** | **_** |
| 17 | **82001324** | Remoção de implante dentário não ósseo integrado | **R$ 222,00** | **_** |
| 17 | **82001367** | Remoção de odontoma | **R$ 268,00** | **_** |
| 17 | **82001510** | Tratamento cirúrgico das fístulas buco nasal com retalho | **R$ 268,00** | **_** |
| 17 | **82001529** | Tratamento cirúrgico das fístulas buco sinusal sem retalho | **R$ 268,00** | **_** |
| 17 | **82001650** | Tratamento de alveolite | **R$ 94,50** | **_** |
| 17 | **82001707** | Ulectomia | **R$ 120,50** | **_** |
| 17 | **82001715** | Ulotomia | **R$ 120,50** | **_** |
| 00 | **89191000** | Remoção de fragmento dentário | **R$ 33,50** | **_** |
| 00 | **89191010** | Colagem de dispositivo ortodôntico | **R$ 60,00** | **_** |
| 17 | **82000344** | Cirurgia Odontologica para aplicação de aloenxertos-  Levantamento de Seio atraumático - Summers | **R$ 326,50** | **_** |
| CRITÉRIOS :  Os procedimentos desta especialidade requerem autorização prévia, à exceção de excisão de ranula, excisão de mucocele, excisão de cálculo salivar, incisão e drenagem de abcesso, reimplante dentário, tratamento de alveolite, controle de hemorragia intra oral, remoção de fragmento dentário.  Os mini implantes só serão avaliados se acompanhados de relatório do ortodontista e a autorização somente será possível para casos ortodônticos e cirúrgicos após auditoria documental inicial.  Caso a cirurgia seja realizada em ambiente hospitalar, ou ambulatorial com sedação, os honorários dos auxiliares serão fixados em 30% para o primeiro auxiliar e 20% para o segundo auxiliar. No caso de cirurgia em ambiente hospitalar os honorários de instrumentador serão fixados em 10% .  Caso seja necessária a anestesia endovenosa, deverá ser encaminhado relatório técnico do Cirurgião  Dentista junto com a solicitação de autorização. O deferimento do pedido de participação de anestesista será incluído na autorização. | | | | |
| **ORTODONTIA** | | | | |
| ***DIAGNÓSTICO*** | | | | |
| 17 | **81000189** | Diagnóstico e planejamento para tratamento odontológico | **R$ 125,50** | **365 DIAS = 1 ANO** |
| 17 | **89200459** | Consulta de urgência ortodontia fixa | **R$ 39,00** | **365 DIAS = 1 ANO** |
| 17 | **89200467** | Consulta de urgência ortodontia móvel | **R$ 39,00** | **_** |
| 17 | **89200475** | Consulta de urgência ortodontia fixa - Horário especial | **R$ 93,50** | **_** |
| 17 | **89200483** | Consulta de urgência ortodontia móvel - Horário especial | **R$ 93,50** | **_** |
| **ORTODONTIA PREVENTIVA** | | | | |
| 17 | **83000097** | Mantenedor de espaço fixo | **R$ 223,00** | **730 DIAS = 2 ANOS** |
| 17 | **83000100** | Mantenedor de espaço removível | **R$ 223,00** | **365 DIAS = 1 ANO** |
| 17 | **86000144** | Arco lingual | **R$ 221,00** | **_** |
| **ORTODONTIA INTERCEPTIVA** | | | | |
| 17 | **85078121** | Aparelho para ronco e apinéia | **R$ 380,00** | **_** |
| 17 | **86000047** | Aparelho de Thurow | **R$ 256,00** | **_** |

| **Table code** | **CÓD. TUSS** | **Descrição TUSS** | **Valor** | **PRAZO INTERVALAR (DIAS)** |
| --- | --- | --- | --- | --- |
| 17 | **86000055** | Aparelho extra-bucal | **R$ 283,00** | **_** |
| 17 | **86000152** | Barra transpalatina fixa | **R$ 229,50** | **_** |
| 17 | **86000160** | Barra transpalatina removível | **R$ 148,00** | **_** |
| 17 | **86000225** | Disjuntor palatino - Hirax | **R$ 346,50** | **_** |
| 17 | **86000233** | Disjuntor palatino - Macnamara | **R$ 346,50** | **_** |
| 17 | **86000314** | Grade palatina fixa | **R$ 235,50** | **_** |
| 17 | **86000322** | Grade palatina removível | **R$ 201,50** | **_** |
| 17 | **86000330** | Herbst encapsulado | **R$ 426,50** | **_** |
| 17 | **86000373** | Manutenção de aparelho ortodôntico - aparelho removível | **R$ 100,00** | **30 DIAS** |
| 17 | **86000381** | Máscara facial – Delaire e Tração Reversa | **R$ 256,00** | **_** |
| 17 | **86000390** | Mentoneira | **R$ 128,50** | **_** |
| 17 | **86000462** | Placa de Hawley | **R$ 160,50** | **_** |
| 17 | **86000470** | 3ª parcela - Placa de Hawley - com torno expansor | **R$ 240,50** | **_** |
| 17 | **86000535** | Placa lábio-ativa | **R$ 222,00** | **_** |
| 17 | **86000560** | Quadrihélice | **R$ 278,50** | **_** |
| **ORTOPEDIA FUNCIONAL DOS MAXILARES** | | | | |
| 17 | **86000020** | Aparelho de Klammt | **R$ 538,50** | **_** |
| 17 | **86000039** | Aparelho de protração mandibular - APM | **R$ 538,50** | **_** |
| 17 | **86000179** | Bionator de Balters | **R$ 538,50** | **_** |
| 17 | **86000365** | Manutenção de aparelho ortodôntico - aparelho ortopédico | **R$ 140,50** | **30 DIAS** |
| 17 | **86000403** | Modelador elástico de Bimler | **R$ 537,00** | **_** |
| 17 | **86000438** | Pistas diretas de Planas - superior e inferior | **R$ 267,50** | **_** |
| 17 | **86000446** | Pistas indiretas de Planas | **R$ 538,50** | **_** |
| 17 | **86000470** | 3ª parcela - Placa de Hawley - com torno expansor | **R$ 240,50** | **_** |
| 17 | **86000500** | Placa de verticalização de caninos | **R$ 201,50** | **_** |
| 17 | **86000527** | Placa encapsulada de Maurício | **R$ 538,50** | **_** |
| 17 | **86000578** | Regulador de função de Franjel | **R$ 538,50** | **_** |
| 17 | **86000586** | Simões Network | **R$ 538,50** | **_** |
| **APARATOLOGIA FIXA** | | | | |
| 17 | **86000098** | Aparelho ortodôntico fixo metálico | **R$ 670,00** | **_** |
| 17 | **86000110** | Aparelho parcial fixo metálico 4 X 2 | **R$ 315,50** | **_** |
| 17 | **86000357** | Manutenção de aparelho ortodôntico - aparelho fixo | **R$ 140,00** | **30 DIAS** |
| 17 | **82001502** | Tracionamento cirúrgico com finalidade ortodôntica | **R$ 201,50** | **_** |
| 17 | **89200513** | 3ª parcela =Placa de HAWLEY sem torno expansor como  complementação final (*) | **R$ 315,50** | **_** |

| **Table code** | **CÓD. TUSS** | **Descrição TUSS** | **Valor** | **PRAZO INTERVALAR (DIAS)** |
| --- | --- | --- | --- | --- |
| Todos os procedimentos destas especialidades requerem autorização prévia.  Somente poderão ser cobradas 2 consultas de urgência no ano, e deve ser enviado relatório técnico de justificativa junto à cobrança. A quantidade de manutenção mensal constará na autorização. Serão autorizadas 12 manutenções, para receber a 13ª o beneficiário deve passar por uma auditoria  intermediária que autorizará mais 12 manutenções. Caso após os 24 meses de tratamento o prestador identifique que o tratamento necessitará de mais tempo, deverá encaminhar relatório justificando a  necessidade para a FIOSAÚDE, que encaminhará o paciente à auditoria . Após a auditoria, se confirmada a necessidade, será concedida nova autorização informando a quantidade de manutenção. Na cobrança de cada manutenção deve ser informado na guia odontológica o número da autorização.  As consultas de urgência horário especial são aquelas cujos atendimentos ocorrem das 22:00h às 07:00h, ou em qualquer horário aos domingos e feriados. SOMENTE PODE SER COBRADA POR CREDENCIADO CONTRATADO PARA SERVIÇO DE URGÊNCIA 24 HORAS.  Para autorização do tratamento ortodôntico o prestador deverá enviar à FIOSAÚDE, parecer contendo diagnóstico da má- oclusão, tipo de tratamento proposto, tipo de nome do aparelho e a previsão de duração.  O valor do orçamento aprovado inclui todos os aparelhos necessários ao tratamento proposto, inclusive os acessórios e os aparelhos de contenção.  Os prazos de tratamento são considerados de forma contínua, mesmo que não sejam realizadas  manutenções. Assim sendo, eventuais interrupções devem ser comunicadas, por escrito, à FIOSAÚDE, para que seja avaliada a possibilidade de suspensão temporária da consultas ortodônticas.  O tracionamento cirúrgico engloba a etapa cirúrgica e a colagem do dispositivo, ou laço para o tracionamento dentário em ortodontia. A PRIMEIRA E SEGUNDA PARCELAS DOS TRATAMENTOS ORTODÔNTICOS E ORTOPÉDICOS TEM O MESMO CÓDIGO E PODEM SER  COBRADAS NUMA ÚNICA GUIA APÓS A AUDITORIA DE COLOCAÇÃO DO APARELHO. A terceira parcela do  Tratamento Ortodôntico ou Ortopedia Funcional dos Maxilares será paga mediante auditoria final, para remoção da aparatologia.  (*) A terceira parcela do Tratamento Ortodôntico Fixo será paga mediante auditoria final, para remoção da aparatologia Somente poderão ser cobradas na Ortodontia Interceptativa a primeira e segunda parcelas da Ortopedia Funcional dos Maxilares. É obrigatório o prestador informar à FIOSAÚDE a ausência do paciente nos tratamentos contínuos. As consultas de manutenção não desmarcadas até 4 horas antes do horário  agendado, que não apresentem justificativas deverão ser atestadas pelo beneficiário. Ao iniciar o tratamento o paciente deverá tomar ciência , por escrito, da possibilidade da cobrança de consultas marcadas e sem comparecimento. | | | | |
| **IMPLANTODONTIA** | | | | |
| 17 | **82000581** | Enxerto com osso autógeno da linha oblíqua | **R$ 436,50** | **_** |
| 17 | **82000603** | Enxerto com osso autógeno do mento | **R$ 436,50** | **_** |
| 17 | **82000620** | Enxerto com osso liofilizado | **R$ 366,00** | **_** |
| 17 | **82000980** | Implante ósseo integrado | **R$ 1.013,50** | **_** |
| 17 | **82001049** | Levantamento do seio maxilar com osso autógeno | **R$ 667,00** | **_** |
| 17 | **82001057** | Levantamento do seio maxilar com osso homólogo | **R$ 667,00** | **_** |
| 17 | **82001065** | Levantamento do seio maxilar com osso liofilizado | **R$ 667,00** | **_** |
| 17 | **82001138** | Reabertura - colocação de cicatrizador | **R$ 462,00** | **_** |
| 17 | **85500062** | Guia cirúrgico para implante | **R$ 190,50** | **180 DIAS** |
| 17 | **85500070** | Intermediário protético (para implantes) | **R$ 468,50** | **1095 DIAS = 3 ANOS** |
| ***CRITÉRIOS :***  **Todos os procedimentos desta especialidade requerem autorização prévia.**  É necessário a cobrança de biomaterial utilizando o código da tabela de biomateriais da FIOSAÚDE. | | | | |
| **ESTOMATOLOGIA** | | | | |
| 17 | **82000026** | Acompanhamento de tratamento/procedimento cirúrgico em  odontologia | **R$ 48,50** | **2 EVENTOS A CADA 30 DIAS** |
| 17 | **82000239** | Biópsia de boca | **R$ 100,00** | **_** |
| 17 | **82000247** | Biópsia de glândula salivar incisional | **R$ 100,00** | **_** |

| **Table code** | **CÓD. TUSS** | **Descrição TUSS** | **Valor** | **PRAZO INTERVALAR (DIAS)** |
| --- | --- | --- | --- | --- |
| 17 | **82000255** | Biópsia de lábio | **R$ 100,00** | **_** |
| 17 | **82000263** | Biópsia de língua | **R$ 100,00** | **_** |
| 17 | **82000271** | Biópsia de mandíbula | **R$ 100,00** | **_** |
| 17 | **82000280** | Biópsia de maxila | **R$ 100,00** | **_** |
| 17 | **82000441** | Coleta de raspado em lesões ou sítios específicos da região  buco-maxilo-facial | **R$ 69,50** | **_** |
| 17 | **82001103** | Punção aspirativa na região buco-maxilo-facial | **R$ 40,00** | **_** |
| 17 | **85045154** | Biópsia excisional | **R$ 272,50** | **365 DIAS = 1 ANO** |
| 00 | **89400016** | Consulta inicial de Estomatologia | **R$ 100,00** | **_** |
| 00 | **89400121** | Teste do azul de Toluidina (*) | **R$ 39,00** | **_** |
| 00 | **89400130** | Sialometria | **R$ 47,50** | **_** |
| **TERAPIA** | | | | |
| 17 | **85400246** | Órtese miorrelaxante (placa oclusal estabilizadora) | **R$ 551,00** | **730 DIAS = 2 ANOS** |
| 00 | **89400156** | Infiltração intra-lesional (por sessão) | **R$ 112,00** | **20 EVENTOS EM 180 DIAS** |
| 00 | **89400164** | Cauterização química (até 5 lesões) | **R$ 112,00** | **_** |
| 00 | **89400172** | Cateterismo de ducto de glândula salivar | **R$ 112,00** | **_** |
| 00 | **89400180** | "Embutimento" de Hiperplasias (por sessão) | **R$ 112,00** | **_** |
| **PROCEDIMENTOS INVASIVOS** | | | | |
| 00 | **89400199** | Remoção de cistos ou tumores intra-ósseos | **R$ 303,00** | **_** |
| 00 | **89400202** | Marsupialização / Micromarsupialização | **R$ 209,00** | **_** |
| ***CRITÉRIOS :***  **Todos os procedimentos desta especialidade requerem autorização prévia.**  (* ) O teste do Azul de Toluidina só será autorizado como método auxiliar na biópsia incisional de lesões ulceradas, leucoplásticas ou eritroplásticas, sugestivas de lesões cancerizáveis ou malignas, ou para o acompanhamento clínico das leucoplasias e/ou eritroplasias. | | | | |
| **CIRURGIA E TRAUMATOLOGIA BUCO-MAXILO-FACIAL** | | | | |
| 00 | **89190445** | Tratamento cirúrgico para osteomielite dos ossos da face | **R$ 378,00** | **_** |
| 00 | **89190461** | Maxilectomia com ou sem esvaziamento orbitário | **R$ 466,50** | **_** |
| 00 | **89190682** | Redução de fratura de ossos próprios do nariz | **R$ 262,50** | **_** |
| 00 | **89190690** | Redução incruenta de fratura unilateral de mandíbula | **R$ 160,00** | **_** |
| 00 | **89190704** | Redução cruenta de fratura unilateral de mandíbula | **R$ 375,00** | **_** |
| 00 | **89190712** | Redução incruenta de fratura bilateral de mandíbula | **R$ 218,00** | **_** |
| 00 | **89190720** | Redução cruenta de fratura bilateral de mandíbula | **R$ 589,50** | **_** |
| 00 | **89190747** | Redução de fratura de côndilo mandibular | **R$ 317,50** | **_** |
| 00 | **89190755** | Fraturas alvéolo-dentárias redução incruenta | **R$ 76,00** | **_** |
| 00 | **89190771** | Redução incruenta de fratura de LE FORT I | **R$ 214,00** | **_** |
| 00 | **89190780** | Redução incruenta de fratura de LE FORT II | **R$ 214,00** | **_** |
| 00 | **89190798** | Redução incruenta de fratura de LE FORT III | **R$ 268,00** | **_** |
| 00 | **89191050** | Ressecção parcial de mandíbula com enxerto ósseo | **R$ 552,50** | **_** |
| 00 | **89191070** | Hemimandibulectomia com colocação de prótese | **R$ 552,50** | **_** |
| 00 | **89191080** | Hemimandibulectomia com enxerto ósseo | **R$ 645,00** | **_** |
| 00 | **89191090** | Mandibulectmia com reconstrução a custa de  osteomiocutâneo | **R$ 962,50** | **_** |
| 00 | **89192000** | Mandibulectmia com reconstrução microcirúrgica | **R$ 1.070,50** | **_** |
| 00 | **89201020** | Excisão com plástica de vermelhão | **R$ 109,00** | **_** |
| 00 | **89201039** | Excisão com reconstrução à custa de retalhos | **R$ 272,50** | **_** |
| 00 | **89201055** | Excisão em cunha | **R$ 128,50** | **_** |
| 00 | **89201071** | Queiloplastia para fissura labial unilateral - por estágio | **R$ 218,00** | **_** |
| 00 | **89201080** | Reconstrução de sulco gengivo-labial | **R$ 294,00** | **_** |
| 00 | **89201098** | Reconstrução total do lábio | **R$ 598,00** | **_** |
| 00 | **89201128** | Reconstrução parcial do lábio | **R$ 294,00** | **_** |

| **Table code** | **CÓD. TUSS** | **Descrição TUSS** | **Valor** | **PRAZO INTERVALAR (DIAS)** |
| --- | --- | --- | --- | --- |
| 00 | **89201280** | Desbridamento cirúrgico - por unidade topográfica (UT) | **R$ 103,00** | **_** |
| 00 | **89201786** | Sutura de extensos ferimentos com ou sem desbridamento | **R$ 47,50** | **_** |
| 00 | **89201794** | Sutura de pequenos ferimentos com ou sem desbridamento | **R$ 47,50** | **_** |
| 00 | **89202132** | Palatoplastia total | **R$ 437,00** | **_** |
| 00 | **89202140** | Plástica do ducto parotídeo | **R$ 294,00** | **_** |
| 00 | **89202234** | Sinusectomia maxilar - via oral (Caldwell-Luc) | **R$ 229,50** | **_** |
| 00 | **89204020** | Excisão de glândula submandibular | **R$ 391,00** | **_** |
| 00 | **89204100** | Ressecção de tumor de glândula sublingual | **R$ 265,50** | **_** |
| 00 | **89207010** | Redução de fratura do malar (sem fixação) | **R$ 218,00** | **_** |
| 00 | **89207029** | Redução de fratura do malar (com fixação) | **R$ 381,50** | **_** |
| 00 | **89207070** | Fratura do arco zigomático - redução cirúrgica com fixação | **R$ 381,50** | **_** |
| 00 | **89207118** | Fratura cominutiva de mandíbula - redução cirúrgica com  fixação óssea e bloqueio intermaxilar eventual | **R$ 481,50** | **_** |
| 00 | **89207134** | Fraturas alveolares - fixação com aparelho e contenção | **R$ 76,00** | **_** |
| 00 | **89207169** | Fratura Lefort I - fixação cirúrgica com síntese óssea,  levantamento e bloqueio intermaxilar eventual | **R$ 214,50** | **_** |
| 00 | **89207177** | Fratura Lefort II - fixação cirúrgica com síntese óssea,  levantamento e bloqueio intermaxilar eventual | **R$ 214,50** | **_** |
| 00 | **89207185** | Fratura Lefort III - fixação cirúrgica com síntese óssea,  levantamento crânio-maxilar e bloqueio intermaxilar eventual | **R$ 268,00** | **_** |
| 00 | **89207193** | Fraturas múltiplas de terço médio da face:fixação cirúrgica  com síntese óssea, levantamento crânio maxilar e bloqueio intermaxilar | **R$ 268,00** | **_** |
| 00 | **89207207** | Fraturas complexas do terço médio da face, fixação cirúrgica  com síntese, levantamento crânio-maxilar, enxerto ósseo, halo craniano | **R$ 375,00** | **_** |
| 00 | **89207215** | Retirada dos meios de fixação (na face) | **R$ 97,50** | **_** |
| 00 | **89207231** | Redução de luxação do ATM | **R$ 262,00** | **_** |
| 00 | **89208017** | Artroplastia para luxação recidivante da articulação têmporo-  mandibular | **R$ 381,50** | **_** |
| 00 | **89208025** | Osteoplastia para prognatismo, micrognatismo ou  laterognatismo | **R$ 375,00** | **_** |
| 00 | **89208050** | Osteotomia tipo Lefort I | **R$ 643,00** | **_** |
| 00 | **89208068** | Osteotomia tipo Lefort II | **R$ 749,00** | **_** |
| 00 | **89208076** | Osteotomia tipo Lefort III - extracraniana | **R$ 776,00** | **_** |
| 00 | **89208106** | Reconstrução parcial da mandíbula com enxerto ósseo | **R$ 529,50** | **_** |
| 00 | **89208112** | Tratamento cirúrgico de anquilose da articulação têmporo  mandibular | **R$ 381,50** | **_** |
| 00 | **89208114** | Reconstrução total de mandíbula com prótese e ou enxerto  ósseo | **R$ 414,00** | **_** |
| 00 | **89209013** | Osteoplastias etmóido orbitais | **R$ 749,00** | **_** |
| 00 | **89209021** | Osteoplastias de mandíbula | **R$ 749,00** | **_** |
| 00 | **89209030** | Osteoplastias do arco zigomático | **R$ 749,00** | **_** |
| 00 | **89209048** | Osteoplastias da órbita | **R$ 749,00** | **_** |
| 00 | **89211042** | Hemimandibulectomia ou ressecção segmentar ou seccional  da mandíbula | **R$ 552,50** | **_** |
| 17 | **87000164** | Sedação consciente com óxido nitroso e oxigênio | **R$ 381,50** | **_** |
| ***CRITÉRIOS :***  **Todos os procedimentos desta especialidade requerem autorização prévia.**  Caso a cirurgia seja realizada em ambiente hospitalar, ou ambulatorial com sedação, os honorários dos auxiliares serão fixados em 30% para o primeiro auxiliar e 20% para o segundo auxiliar. No caso de cirurgia em ambiente hospitalar, os honorários de instrumentador serão fixados em 10% .  A Sedação com óxido nitroso somente poderá ser feita por prestador habilitado e cadastrado na FIOSAÚDE. | | | | |

| **Appendix IV -** Procedures considered for cost estimates based on the FioSaúde table. | | |
| --- | --- | --- |
| Procedure | Code FioSaúde | Cost * |
| Initial dental appointment | 81000065 | R$ 38,00 |
| Fluoride varnish topical application (per dental arch) | 84000090 | R$ 19,50 |
| Oral health education | 84000139 | R$ 28,00 |
| Biofilm control (dental plaque) | 84000163 | R$ 25,50 |
| Prophylaxis | 84000198 | R$ 27,00 |
| Glass ionomer restoration (regardless the number of faces) | 85100137 | R$ 61,00 |
| Hall technique restoration on a deciduous tooth | 83000046 | R$ 136,00 |
| Simple extraction of deciduous tooth | 83000089 | R$ 54,50 |

* Costs in Brazilian Reais. On 20 August 2024, 1 US Dollar could buy 5.5 Reais.

| **Appendix V** - Costs related to events in the proposed model. | | |
| --- | --- | --- |
| Events | Costs | Total Cost |
| No cavity/ Previous cavity | Initial dental appointment + Oral health education + Biofilm control + Prophylaxis | R$ 118,50 |
| Glass ionomer restoration | Initial dental appointment + Oral health education + Biofilm control + Prophylaxis + Glass ionomer restoration | R$ 179,50 |
| Hall technique restoration | Initial dental appointment + Oral health education + Biofilm control + Prophylaxis + Hall technique restoration | R$ 254,50 |
| Tooth Extraction | Initial dental appointment + Oral health education + Biofilm control + Prophylaxis + Tooth Extraction | R$ 173,50 |
| Fluoride varnish topical application * | Fluoride varnish topical application | R$ 78,00 |

*Value added in all events in the FV application
